# A systematic review of reliability and objective criterion-related validity of physical activity questionnaires

**DOI:** 10.1186/1479-5868-9-103

**Published:** 2012-08-31

**Authors:** Hendrik JF Helmerhorst, Søren Brage, Janet Warren, Herve Besson, Ulf Ekelund

**Affiliations:** 1 Medical Research Council Epidemiology Unit, Cambridge, UK; 2 Academic Medical Center, University of Amsterdam, Amsterdam, The Netherlands; 3 Medical Research Council Human Nutrition Resource centre, Cambridge, UK; 4 Danone Baby Nutrition (Nutricia Ltd), Trowbridge, UK; 5 Department of Sports Medicine, Norwegian School of Sport Sciences, Oslo, Norway

**Keywords:** Systematic review, Physical activity, Self-report, Accelerometry, Validity, Reliability

## Abstract

Physical inactivity is one of the four leading risk factors for global mortality. Accurate measurement of physical activity (PA) and in particular by physical activity questionnaires (PAQs) remains a challenge. The aim of this paper is to provide an updated systematic review of the reliability and validity characteristics of existing and more recently developed PAQs and to quantitatively compare the performance between existing and newly developed PAQs.

A literature search of electronic databases was performed for studies assessing reliability and validity data of PAQs using an objective criterion measurement of PA between January 1997 and December 2011. Articles meeting the inclusion criteria were screened and data were extracted to provide a systematic overview of measurement properties. Due to differences in reported outcomes and criterion methods a quantitative meta-analysis was not possible.

In total, 31 studies testing 34 newly developed PAQs, and 65 studies examining 96 existing PAQs were included. Very few PAQs showed good results on both reliability and validity. Median reliability correlation coefficients were 0.62–0.71 for existing, and 0.74–0.76 for new PAQs. Median validity coefficients ranged from 0.30–0.39 for existing, and from 0.25–0.41 for new PAQs.

Although the majority of PAQs appear to have acceptable reliability, the validity is moderate at best. Newly developed PAQs do not appear to perform substantially better than existing PAQs in terms of reliability and validity. Future PAQ studies should include measures of absolute validity and the error structure of the instrument.

## Background

Physical inactivity is considered to be one of the four leading risk factors for global mortality [1]. The measurement of physical activity is a challenging and complex procedure. Valid and reliable measures of physical activity (PA) are required to: document the frequency, duration and distribution of PA in defined populations; evaluate the prevalence of individuals meeting health recommendations; examine the effect of various intensities of physical activity on specific health parameters; make cross-cultural comparisons and evaluate the effects of interventions [2].

Physical activity questionnaires (PAQs) are often the most feasible method when assessing PA in large-scale studies, likely because of their low cost and convenience but these instruments have limitations and should be selected and used judiciously. PAQs are prone to measurement error and bias due to misreporting, either deliberate (social desirability bias) or because of cognitive limitations related to recall or comprehension [3,4]. Cognitive immaturity or degeneration can make self-report of physical activity particularly difficult in the young and elderly [5,6]. Despite more frequent use of objective assessment methods to measure physical activity, PAQs still provide a practical method for PA assessment in surveillance systems, for risk stratification and when examining etiology of disease in large observational studies. Most PAQs are designed to be able to measure multiple dimensions of PA by reporting type, location, domain and context of the activity, provide estimates of time spent in activities of various levels of intensity, and may be able to rank individuals according to intensity levels of reported activity [7,8]. However, results from studies aimed at evaluating the validity of PAQs assessed in one population cannot be systematically extrapolated to other populations, ethnic groups, or other geographical regions. Consequently, a great variety of PAQs have been developed and tested for reliability and validity in recent years.

A comprehensive review of PAQs for use in adults was published in 1997 [9]. Since then, reviews summarizing the validity and reliability of PAQs have been carried out in children [10-12] and preschoolers [13]. Recently, specific reviews were published assessing the quality of PAQs available for children [11], adults [14] and the elderly [15]. The aim of the present study was to systematically review the literature on reliability of PAQs as well as their validity evaluated against objective criterion methods, for use in all age groups, published between January 1997 and December 2011 to quantitatively compare the performance between existing and newly developed PAQs.

## Methods

### Inclusion criteria

Studies meeting all of the following inclusion criteria were included: (i) published in the English language between January 1997 and December 2011; (ii) self- or interviewer-administered PAQs or parental proxy reports reporting both reliability and validity results; (iii) PAQs reporting validity results only, when the reliability data has been published previously; (iv) PAQs developed for a healthy general population and for observational surveillance studies; (v) PAQs tested in its original form or in an adapted version if results were reported for validity and reliability or validity only, when reliability results were published before; (vi) validity tested against an objective criterion measure of PA (i.e. accelerometry, heart rate, combined heart rate and accelerometry, doubly labeled water (DLW)); (vii) results on validity obtained by pedometer where the questionnaire was specifically developed to assess walking only.

### Exclusion criteria

We excluded studies that reported: (i) reliability and validity results in groups with specific clinical or medical conditions (except pregnancy); (ii) results from PAQs that were designed for specific intervention studies; (iii) results where the validity of the PAQ was tested against another self-report method (i.e. diaries, logs); (iv); results on validity using pedometers (except if walking only was tested) and indirect measures of physical activity (e.g. VO_2max_ and body composition); (v) results on essential adaptations of original PAQs, without any published results on both reliability and validity.

### Literature search

The PubMed, Medline and Web of Science databases were systematically searched using the following lists and terms:

List A: (physical activity AND health survey OR population survey OR question*)

List B: List B: measure* (i.e. measures, measurement), assess* (i.e. assessment, assessed), self-report, exercise, valid* (i.e. valid, validation, validity), reliab* (i.e. reliable, reliability), reproducible, accelerometer, heart rate, doubly labelled water, doubly labeled water. The search included titles, abstracts, key words and full texts.

Key search terms in List A were combined with each of the terms in List B.

**Figure 1:**
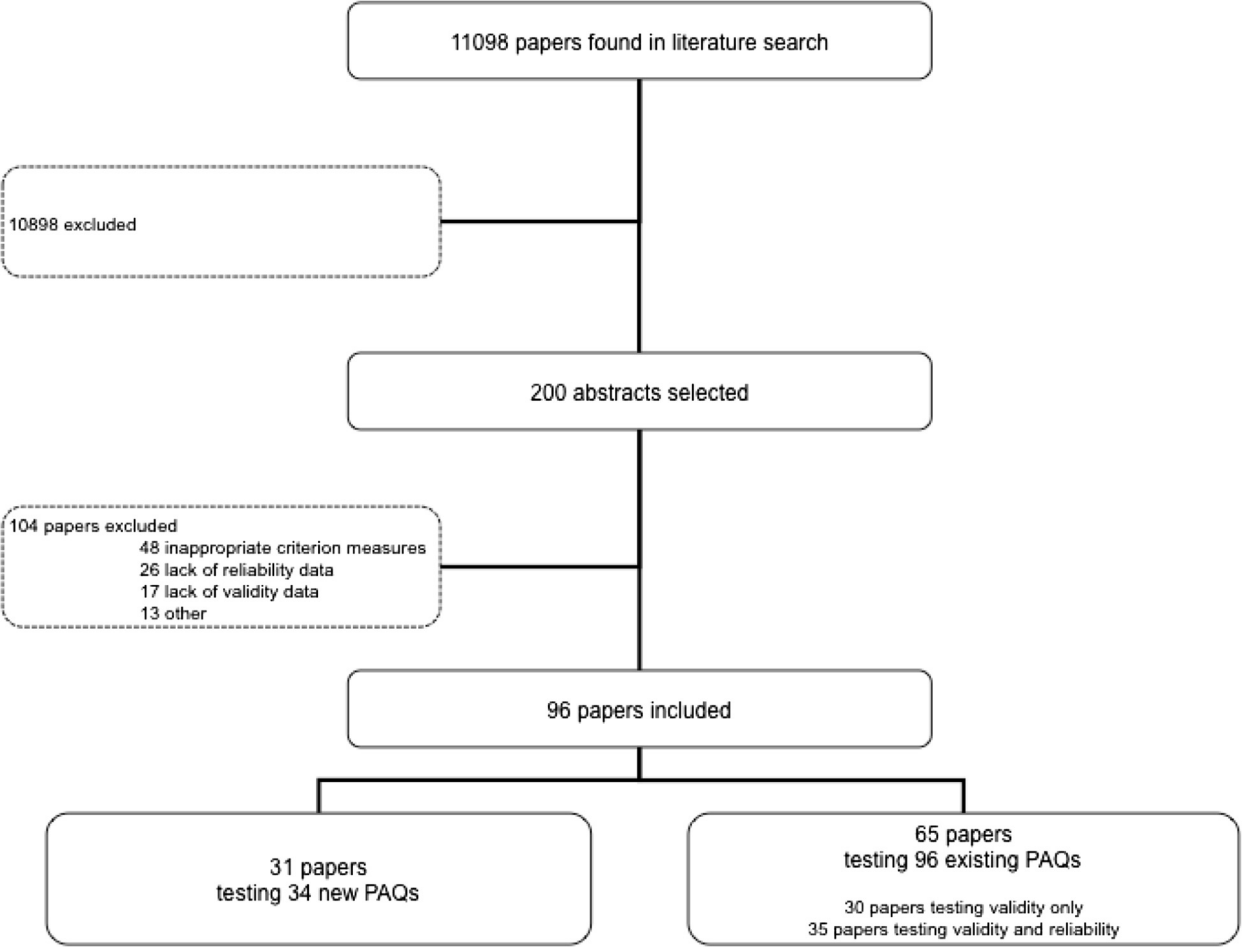
Overview of the literature search.

The literature search was undertaken in two stages. The original literature search (1997–2008) was undertaken by two of the authors (JW, HB) independently and search results were compared and verified. The literature search was then updated to include studies up to December 2011 using exactly the same search criteria (HH). A second search strategy included screening references lists of publications that matched the inclusion criteria and any other publications of which the authors were aware but did not show up during the original literature search. Figure 1 displays an overview of the literature search.

### Data collection and extraction

Data were extracted using a standardized pro-forma which included sample characteristics, questionnaire details, methods of validity and reliability testing, test results and authors’ conclusions. We retrieved full text of articles of all abstracts that met our inclusion criteria. Any queries about the inclusion of papers were resolved by one of the authors (UE).

### Reliability

Reliability in all studies was tested through a test-retest procedure to measure consistency of the PAQs. Reliability results from included studies were reported as: intraclass correlation coefficients (ICC); Pearson and Spearman correlation coefficients; and agreement measures using Cohen’s weighted kappa (κ) and mean differences. Reliability was considered poor, moderate (acceptable), or strong when correlation coefficients or kappa statistics were <0.4, 0.4–0.8 or >0.8, respectively [16]. Similarly, an ICC > 0.70 or >0.90 was considered as acceptable and strong, respectively, in those studies reporting this measure [17].

Medians of reliability correlation coefficients across studies were calculated and included in the tables when possible.

### Validity

Correlation coefficients were the most commonly used measures of validity, although the Bland-Altman technique [18] which determines absolute agreement between two measures expressed in the same units, was also frequently used. The Bland-Altman method estimates the mean bias and the 95 % limits of agreement (± 2SD of the difference) and is usually plotted as the difference between the methods against the mean of the methods for visual inspection of the error pattern throughout the measurement range; the dependence of error with the underlying level can be summarised in the error correlation coefficient but this was only seldom reported.

Medians of included validity correlation coefficients were calculated and included in the tables when possible. When calculating the medians, we excluded those studies reporting correlation coefficients for the associations of self-reported sedentary time. The medians for sedentary time are reported separately and associations of sedentary time with measures of total physical activity (i.e. total energy expenditure [TEE], physical activity level [PAL] and total activity from accelerometry [mean counts]) from the criterion method were excluded in these analyses as these measures are expected to be inversely related.

### Classification

Questionnaires were classified as new or existing (i.e. previously published test results) PAQ. Existing questionnaires were subdivided into those which reported new reliability and validity results, and those which reported new results on validity only but had previously reported results on reliability. Questionnaires were classified as new, when the concerning study was the first to publish reliability and objective validity data on the PAQ. Hereafter, studies were further stratified for age group of the sample. Study populations with a mean age lower than 18 years were categorised as youth, 18 – 65 years were classified as adults, and elderly above 65 years.

### PAQs included

PAQ abbreviations are listed in Table 1, with their respective timeframe. The details of these studies are shown in Tables 2 (new PAQs) and 5 (existing PAQs). A range of tests were used to assess reliability and validity with some studies reporting results for a total questionnaire summary score, and others assessing reliability and validity for various aspects, intensities, or domains of the questionnaire and/or by subgroups within the test population. The total score or index for the PAQ was reported, if available. In the absence of a total score, correlation coefficients by intensity category or group are reported. Where multiple results were reported, a decision was made about the data that constituted the main results based on the stated objectives for the study or questionnaire. Several studies compared results to another questionnaire concurrently but if this was a secondary aim of the specific study, the results were not included.

**Table 1:** List of questionnaire abbreviations and the corresponding definitions

**Acronym**	**Definition**	**Timeframe**
1WPAR	One-week Physical Activity Recall	Last 7 days
7DPAR	7-Day Physical Activity Recall	Last 7 days
7DR	7-Day Recall	Last 7 days
7DR-O	7-Day Recall (occupational activity)	Last 7 days
AAFQ	Arizona Activity Frequency Questionnaire	Last 28 days
AAS	Active Australian Survey (modified version)	Last 7 days, usual week
Activitygram	Activitygram	Last 3 days
AQuAA	Activity Questionnaire for Adolescents and Adults	Last 7 days
AWAS	Australian Women's Activity Survey	Typical week last month
BAD	Bouchard Activity Diary	Last 3 days
BAQ	Baecke Activity Questionnaire	Usual activity
BAQ-mod	Baecke Activity Questionnaire (modified version)	Last year
BONES PAS	Beat Osteoporosis: Nourish and Exercise Skeletons Physical Activity Survey	Last 2 days
BRFSS PAQ	Behavioral Risk Factor Surveillance System Physical Activity Questionnaire (2001 version)	Typical week
CAPS-4WR	Cross-Cultural Activity Participation Study – 4 Weeks activity Recall	4 weeks
CAPS-TWR	Cross-Cultural Activity Participation Study – Typical Week activity Recall	Typical week
CAQ	College Alumnus Questionnaire	Last 7 days
CAQ-PAI	College Alumnus Questionnaire – Physical Activity Index	Last 7 days
CDPAQ	Computer Delivered Physical Activity Questionnaire	Previous day
CHAMPS	Community Healthy Activities Model Program for Seniors	Typical week last month
CHAMPS-MMSCV	Community Healthy Activities Model Program for Seniors (Modified Mailed Self-Complete Version)	Last 7 days
CHASE	Child Heart and Health Study in England questionnaire	Typical week
CLASS	Children's Leisure Activity Study Survey questionnaire	Typical week
CPAQ	Children's Physical Activity Questionnaire	Last 7 days
DQ-mod	Dallosso Questionnaire (modified version)	Typical day last week, typical week
EPAQ	EPIC Physical Activity Questionnaire	Last year
EPAQ-s	EPIC Physical Activity Questionnaire (short version)	Last year
EPAQ2	EPIC Physical Activity Questionnaire (second version)	Last year
FCPQ	Five City Project Questionnaire	Typical week
Fels PAQ	Fels Physical Activity Questionnaire for children	Last year
FPACQ	Flemish Physical Activity Computerized Questionnaire	Typical week
GAQ	GEMS (Girls Health Enrichment Multi-site Studies) Activity Questionnaire	Previous day, usual activity
GLTEQ	Godin Leisure-Time Exercise Questionnaire	Typical week
GPAQ	Global Physical Activity Questionnaire	Typical week
GSQ	Godin-Shephard Questionnaire	Typical week
HAQ	Harvard Alumni Questionnaire	Typical week
HBSC	Health Behaviour in School Children Questionnaire	Typical week
HEPA99	Swiss Health Enhancing Physical Activity Survey 1999	Typical week
HUNT1	Nord-Trøndelag Health Study questionnaire (version 1)	Last 7 days
HUNT2	Nord-Trøndelag Health Study questionnaire (version 2)	Last year
IPAQ	International Physical Activity Questionnaire	Last 7 days, typical week
IPAQ-A	International Physical Activity Questionnaire (modified for Adolescents)	Last 7 days
IPAQ-E	International Physical Activity Questionnaire (short version modified for Elderly)	Last 7 days
IPAQ-LC	International Physical Activity Questionnaire (Long version in Chinese)	Last 7 days
IPAQ-s	International Physical Activity Questionnaire (short version)	Last 7 days
IPAQ-SALVCF	International Physical Activity Questionnaire (Self-Administered Long Version in Canadian French)	Last 7 days
JPAC	Jackson heart Physical Activity Cohort (i.e. modified KPAS)	Last year
KPAS	Kaiser Physical Activity Survey	Last year
KPAS-mod	Kaiser Physical Activity Survey (modified version)	Current trimester
LRC	Lipid Research Clinics questionnaire	Usual activity
MAQ	Modifiable Activity Questionnaire	Last year
MARCA	Multimedia Activity Recall for Children and Adolescents	Previous day
MLTPAQ	Minnesota Leisure Time Physical Activity Questionnaire	Last year
MRPARQ	Many Rivers Physical Activity Recall Questionnaire	Typical week
NHS-PAQ	Nurses' Health Study II – Physical Activity Questionnaire	Last 7 days
OIMQ	Office In Motion Questionnaire	Last 7 days
OPAQ	Occupational Physical Activity Questionnaire	Typical week
PAAT	Physical Activity Assessment Tool	Last 7 days
PAQ-A	Physical Activity Questionnaire for Adolescents	Last 7 days
PAQ-C	Physical Activity Questionnaire for older Children	Last 7 days
PAQ-EJ	Physical Activity Questionnaire for Elderly Japanese	Typical week last month
PASE	Physical Activity Scale for the Elderly	Last 7 days
PDPAR	Previous Day Physical Activity Recall	Previous day
PMMAQ	Past Month – Modifiable Activity Questionnaire	Last month
PPAQ	Pregnancy Physical Activity Questionnaire	Current trimester
Pre-PAQ	Preschool-age Children's Physical Activity Questionnaire	Last 3 days (1 week, 2 weekend days)
PWMAQ	Past Week – Modifiable Activity Questionnaire	Last 7 days
PYTPAQ	Past Year Total Physical Activity Questionnaire	Last year
QAPSE	Questionnaire d'Activité Physique Saint-Etienne	Typical week last year
RPAQ	Recent Physical Activity Questionnaire (i.e. EPAQ2 redesigned)	Last month
RPAR	Recess Physical Activity Recall	Last recess
S7DR	Stanford 7-Day Recall	Last 7 days
SAPAC	Self-Administered Physical Activity Checklist (modified version)	Last 3 days
SBQ	Sedentary Behavior Questionnaire	Typical week
SHAPES	School Health Action, Planning Evaluation System	Last 7 days
SHS97	Swiss Health Survey 1997	Typical week
SP2PAQ	Singapore Prospective Study Program Physical Activity Questionnaire	Last 3 months
SPAQ	Scottish Physical Activity Questionnaire	Last 7 days
SSAAQ	Sub-Saharan Africa Activity Questionnaire	Last year
SUA	Stanford Usual Activity	Usual activity, last 3 months
SWAPAQ	Swedish Adolescent Physical Activity Questionnaire	Last 7 days
TCQ	Tecumseh Community Questionnaire	Last year
TOQ	Tecumseh Occupational Questionnaire	Last 7 days
WAC	Weekly Activity Checklist	Last 7 days
WHI-PAQ	Women's Health Initiative – Physical Activity Questionnaire	Last 7 days
YMCLS	Youth Media Campaign Longitudinal Survey	Last 7 days
YPAQ	Youth Physical Activity Questionnaire	Last 7 days, previous day
YPAS	Yale Physical Activity Scale	Typical week last month
YRBS	Youth Risk Behavior Survey	Last 7 days
*PAEE*	*Physical Activity Energy Expenditure*	
*TEE*	*Total Energy Expenditure*	
*MPA*	*Moderate intensity Physical Activity*	
*VPA*	*Vigorous intensity Physical Activity*	
*MVPA*	*Moderate and Vigorous intensity Physical Activity*	
*PAL*	*Physical Activity Level*	
*MET*	*Metabolic Equivalent of Task*	
*Acc*	*Accelerometry*	
*HR*	*Heart Rate monitoring*	
*DLW*	*Doubly Labeled Water*	
*Ped*	*Pedometer*	
*ML*	*Mini-Logger*	

Frequently used acronyms also included at the bottom of the table.

**Table 2:** Descriptive characteristics of new PAQs

**Age group**	**Reference**	**Name questionnaire**	**Country**	**Domains of activity**	**Population**				**Primary outcome**
					***Size***	***Age (years)***	***Sex***	***Ethnicity***	
Youth	Dwyer (2011)[19]	Pre-PAQ	Australia	Habitual and sedentary activities in home environment	103 reliability, 67 validity	3 - 5.9	M/F	Mainly Caucasian	Min/day
Youth	Economos (2010)[20]	BONES PAS	United States	Common activities for children	41 reliability, 40 validity	6 - 9	M/F	–	METs, WBF score
Youth	Martinez-Gomez (2010)[21]	RPAR	Spain	Sedentary, leisure, transportation, sports/exercise	125	12 - 14	M/F	–	MET-min, minutes
Youth	Philippaerts (2006)[22]	FPACQ	Belgium	Sedentary, leisure, occupation, transportation	33	12 - 18	M/F	Mainly Caucasian	Total hr/week, METs
Youth	Ridley (2001)[23]	CDPAQ	Australia	Type, duration, intensity, organization of activities before, during and after school	30	11.96 ± 0.53	M/F	–	METs, minutes
Youth	Ridley (2006)[24]	MARCA	Australia	Sedentary, leisure, household, occupation, transportation, sports/exercise during a school day or another day	32 reliability, 66 validity	9 - 15	M/F	–	PAL, EE, total time in any activity
Youth	Telford (2004)[25]	CLASS	Australia	30 physical activities over weekdays and weekends	280	5 - 6, 10 - 12	M/F	Mainly Australian born	Total min/week
Youth	Treuth (2003)[26]	GAQ, Activitygram	United States	GAQ: 28 physical, 7 sedentary usual activities. Activitygram: log of all activities in light, moderate, vigorous intensity	68	8 - 9	F	African-American	GAQ score, Activitygram score
Youth	Treuth (2005)[27]	Fels PAQ	United States	Leisure, occupation, sports/exercise	229	7 - 19	M/F	–	Fels PAQ scores
Youth	Welk (2007)[28]	YMCLS	United States	Free time activity, organized activity, any outside school activity	192	9 - 13	M/F	Mixed	Frequency/week, min/day
Youth	Wong (2006)[29]	SHAPES	Canada	Moderate and vigorous activity and participation in physical, sedentary activities	1636 reliability, 67 validity	Grades 6 - 12	M/F	Mixed	Min/day, EE
Adults	Ainsworth (2000)[30]	KPAS	United States	Household, occupation, sports/exercise, active living habits	50	20 - 60	F	Mainly white	KPAS activity indexes
Adults	Besson (2010)[31]	RPAQ	United Kingdom	Sedentary, leisure, household, occupation, transportation	131 reliability, 50 validity	21 - 55	M/F	–	MET-hr/day, PAEE (kJ/day), TEE (kJ/day)
Adults	Chasan-Taber (2004)[32]	PPAQ	United States	Sedentary, household, occupation, transportation, sports/exercise	63	16 - 40	F	Mixed	MET-hr/week
Adults	Chinapaw (2009)[33]	AQuAA	Netherlands	Sedentary, leisure, household, occupation, transportation, sports/exercise	111 reliability, 89 validity	12 - 38	M/F	–	MET-min/week, AQuAA score
Adults	Craig (2003)[34]	IPAQ	12 countries	Short form: sitting, walking, moderate and vigorous intensity. Long form: sedentary, leisure, household, occupation, transportation	Long form: 1880 reliability, 744 validityShort form: 1974 reliability, 781 validity.	18 - 65	M/F	Mixed	Weighted MET-min/week
Adults	Fjeldsoe (2009)[35]	AWAS	Australia	Sedentary, household, occupation, transportation, planned activities	40 reliability, 75 validity	32 ± 5	F	–	Total min/week for each intensity level
Adults	Friedenreich (2006)[36]	PYTPAQ	Canada	Leisure, household, occupation	154	35 - 65	M/F	–	MET-hr/week, total hours/week
Adults	Kurtze (2007)[37]	HUNT2	Norway	Leisure, occupation in light and hard intensity	108	20 - 39	M	–	Light, hard PA summary score
Adults	Kurtze (2008)[38]	HUNT1	Norway	Leisure	108	20 - 39	M	–	Summary index of weekly PA
Adults	Lowther (1999)[39]	SPAQ	Scotland	Leisure, occupation in moderate, hard, very hard intensity	34 reliability, 30 validity	33 ± 12, 33 ± 11 (reliability); 37 ± 11, 35 ± 14 (validity)	M/F	–	Total min/week
Adults	Mäder (2006)[40]	SHS97, HEPA99, IPAQ, OIMQ	Switzerland	Sedentary, leisure, household, occupation, transportation	178 reliability, 35 validity	15 - 75	M/F	Mainly Caucasian	MET-min/week, days/week, combined variable
Adults	Meriwether (2006)[41]	PAAT	United States	Leisure, household, occupation, transportation	68 reliability, 63 validity	20 - 61	M/F	Mainly white	Total min/week
Adults	Reis (2005)[42]	OPAQ	United States	Occupational sitting/standing, walking, heavy labour	41	20 - 63	M/F	–	MET-min/week
Adults	Rosenberg (2010)[43]	SBQ	United States	9 sedentary activities	49 reliability, 842 validity	20.4 ± 1.3 (reliability); ♀41.2 ± 8.7, ♂43.9 ± 8.0 (validity)	M/F	Mainly white	Total hr/week
Adults	Sobngwi (2001)[44]	SSAAQ	Cameroon	Leisure, occupation, walking/cycling	89 reliability, 54 acc, 89 HR	19 - 68	M/F	African	Total hr/day, MET-hr/day
Adults	Timperio (2003)[45]	1WPAR	Australia	All activities in walking, moderate, vigorous intensity	118 reliability, 122 validity	25 - 47	M/F	–	MET-min/day
Adults	Wareham (2002)[46]	EPAQ2	United Kingdom	Sedentary, leisure, household, occupation, transportation	399 reliability, 173 validity	40 - 74	M/F	Mixed	MET-hr/week
Adults	Wareham (2003)[47]	EPAQ-s	United Kingdom	Leisure, household, occupation, transportation	2271 reliability, 173 validity	40 - 74	M/F	Mixed	PA index, mean day PAR
Adults	Yore (2007)[48]	BRFSS PAQ (2001 version)	United States	Leisure, household, occupation, transportation	60	44.5 ± 15.7	M/F	Mixed	MPA and VPA min/week
Elderly	Yasunaga (2007)[49]	PAQ-EJ	Japan	Household, occupation, transportation, sports/exercise	147	65 - 85	M/F	Japanese	PAQ-EJ score (MET-hr/week)

Domains named in paper were reclassified, unless the activities were very different from categories used, according to the following system: Occupation: work, school, labour*.* Transportation: travel, commuting, employment*.* Household: home/life, housework, caregiving, domestic life, child/elder/self care, cooking, chores, gardening, stair climbing. Leisure: leisure, recreation time. Sports/exercise: play, sports, exercise, workout. Sedentary: sedentary behaviours, e.g. sitting, TV viewing activities, eating, sleeping, bathing, inactivity. *"– = not stated, M = Male, F = Female.*

Results were reported for both total score and other aspects (e.g. domain, intensity) when this substantially added to the information for the specific study, for example when total PA was tested against a different validation method than PA intensities [31]. Some questionnaires assessed sedentary behaviour and these results are specifically reported in the tables or text. Sedentary behaviour has recently been suggested to be considered distinctively from physical activity in associations with health outcomes [50].

## Results

The search string (JW and HH) resulted in a total of 11098 hits. The first literature search resulted in 125 papers being retrieved for data extraction. The update of the literature review to December 2011 resulted in a further 75 papers being retrieved for data extraction (Figure 1). More than half of the papers retrieved were excluded (n = 104). The main reasons for exclusion were inappropriate criterion measures, generally a measure of aerobic fitness (n = 48), and lack of information on reliability (n = 26) or validity (n = 17) (Figure 1).

### New PAQs

The description of newly developed PAQs is summarized in Table 2. The literature search found 31 articles, reporting results from 34 newly developed PAQs of which 10 were from the United States, 10 from Europe, six from Australia, two from Canada, and one study from Japan and Sub-Saharan Africa, respectively. Of note was a 12–country international study testing the International Physical Activity Questionnaire (IPAQ) [34]. This questionnaire is available in a short form for surveillance and in a longer form when more detailed physical activity information is collected. Both forms are available in a number of languages. IPAQ has been rigorously tested for reliability and validity and this has been replicated in a number of countries.

Nineteen studies tested the reliability and validity in adults, an additional 11 studies focused on youth [19-29] and one study was performed in Japanese elderly (n = 1) [49]. Most studies (n = 25) included men and women, four studies [26,30,32,35] reported data in women and two studies [37,38] in men only. The number of participants varied from 30 to 2271, and several studies [19,20,29,31,33-35,39-41,43-47] performed reliability testing in a larger sample than their test of criterion validity. The most common response timeframe was the last seven days, with seven studies [27,30,36,37,44,46,47] using a timeframe covering the last year (Table 1). All PAQs captured some elements of leisure time and recreational activity, although most questionnaires also addressed multiple domains of activity. Sedentary time is also a commonly captured behaviour from the newly developed questionnaires and has been given some extra attention in recent publications and in the current results. Several recent PAQs, such as the EPIC Physical Activity Questionnaire (EPAQ2) and the Recent Physical Activity Questionnaire (RPAQ), aim to measure the totality of physical activity by domains [31,46,47,51]. The final outcome of the majority of PAQs was reported as time-integrated MET values, e.g. MET-min/week.

### Reliability

All reliability results for new PAQs are listed in Table 3.

**Table 3:** Reliability results of new PAQs

**Age Group**	**Reference**	**Test-retest period**	**PAQ**	**Variables tested**	**Reliability results**	
					***Correlation coefficients***	***Agreement***
Youth	Dwyer (2011)[19]	1 - 2 weeks	Pre-PAQ	Level 5 min/day(Q1) – level 5 min/day(Q2)	ICC = 0.64	–
				Level 4 min/day(Q1) – level 4 min/day(Q2)	ICC = 0.44	–
				Level 3 min/day(Q1) – level 3 min/day(Q2)	ICC = 0.53	–
				Levels 1–2 min/day(Q1) – levels 1–2 min/day(Q2)	ICC = 0.44	–
Youth	Economos (2010)[20]	1 - 2 hours	BONES PAS	High METs(Q1) – high METs(Q2)	Spearman r (95 % CI) = 0.57 (0.32;0.75), P < 0.001	–
				Moderate-high METs(Q1) – moderate-high METs(Q2)	Spearman r (95 % CI) = 0.74 (0.56;0.85), P < 0.001	–
				WBF score(Q1) – WBF score(Q2)	Spearman r (95 % CI) = 0.71 (0.51;0.83), P < 0.001	–
Youth	Martinez-Gomez (2010)[21]	1 hour	RPAR	Total MET-min(Q1) – total MET-min(Q2)	ICC = 0.87	–
Youth	Philippaerts (2006)[22]	9 days	FPACQ	Total hr/week(Q1) – total hr/week(Q2)	ICC = 0.68	κ = 0.50
				Total EE(Q1) – total EE(Q2)	ICC = 0.80	κ = 0.53
				Inactivity(Q1) – inactivity(Q2)	ICC = 0.83	κ = 0.61
Youth	Ridley (2001)[23]	7 days	CDPAQ	Total METs(Q1) – total METs(Q2)	ICC = 0.98 (P < 0.05)	–
				Total min(Q1) – total min(Q2)	ICC = 0.91 (P < 0.05)	–
			CDPAQ-HC	Total METs(Q1) – total METs(Q2)	ICC = 0.98 (P < 0.05)	–
				Total min(Q1) – total min(Q2)	ICC = 0.96 (P < 0.05)	–
Youth	Ridley (2006)[24]	Within 24 hours	MARCA	PAL(Q1) – PAL(Q2)	ICC = 0.93	95 % LoA = −0.30 – 0.30
Youth	Telford (2004)[25]	> 14 days	CLASS-parental report	5-6 yrs: frequency(Q1) – frequency(Q2)	ICC = 0.83 (P < 0.001)	–
				10-12 yrs: frequency(Q1) – frequency(Q2)	ICC = 0.69 (P < 0.001)	–
				5-6 yrs: duration(Q1) – duration(Q2)	ICC = 0.76 (P < 0.001)	–
				10-12 yrs: duration(Q1) – duration(Q2)	ICC = 0.74 (P < 0.001)	–
			CLASS-self	10-12 yrs: frequency(Q1) – frequency(Q2)	ICC = 0.36 (P < 0.01)	–
				10-12 yrs: duration(Q1) – duration(Q2)	ICC = 0.24	–
Youth	Treuth (2003)[26]	4 days	GAQ	Yesterday: GAQ score(Q1) – GAQ score(Q2)	Pearson r = 0.7833 (P < 0.0001)	–
				Usual: GAQ score(Q1) – GAQ score(Q2)	Pearson r = 0.8187 (P < 0.0001)	–
				Yesterday: TV watching(Q1) – TV watching(Q2)	Pearson r = 0.3454 (P = 0.0043)	–
				Usual: TV watching(Q1) – TV watching(Q2)	Pearson r = 0.3827 (P = 0.0015)	–
				Yesterday: other sedentary(Q1) – other sedentary(Q2)	Pearson r = 0.4695 (P < 0.0001)	–
				Usual: other sedentary(Q1) – other sedentary(Q2)	Pearson r = 0.4837 (P < 0.0001)	–
		3 days	Activitygram	Activitygram score(Q1) – activitygram score(Q2)	ICC = 0.24 (P = 0.005)	–
Youth	Treuth (2005)[27]	6 days	Fels PAQ	Girls: Fels PAQ score(Q1) – Fels PAQ score(Q2)	ICC = 0.67	–
				Boys: Fels PAQ score(Q1) – Fels PAQ score(Q2)	ICC = 0.65	–
Youth	Welk (2007)[28]	7 days	YMCLS	Total activity(Q1) – total activity(Q2)	ICC (95 % CI) = 0.60 (0.47;0.70)	–
Youth	Wong (2006)[29]	7 days	SHAPES	Combined activity(Q1) – combined activity(Q2)	–	κ (±SD) = 0.58 ± 0.17
				Sedentary activity(Q1) – sedentary activity(Q2)	–	κ (±SD) = 0.55 ± 0.01
Adults	Ainsworth (2000)[30]	1 month	KPAS	3-point summary index(Q1) – 3-point summary index(Q2)	ICC = 0.82 (P < 0.0001)	–
				4-point summary index(Q1) – 4-point summary index(Q2)	ICC = 0.83 (P < 0.0001)	–
Adults	Besson (2010)[31]	± 2 weeks	RPAQ	PAEE(Q1) – PAEE(Q2)	ICC = 0.76 (P < 0.001)	–
				Sedentary time(Q1) – sedentary time(Q2)	ICC = 0.76 (P < 0.001)	–
Adults	Chasan-Taber (2004)[32]	7 days	PPAQ	Total activity(Q1) – total activity(Q2)	ICC = 0.78	–
				Sedentary(Q1) – sedentary(Q2)	ICC = 0.79	–
Adults	Chinapaw (2009)[33]	2 weeks	AQuAA	Adolescents: AQuAA score(Q1) – AQuAA score(Q2)	ICC (95 % CI) = 0.44 (0.16;0.65)	–
				Adults: AQuAA score(Q1) – AQuAA score(Q2)	ICC (95 % CI) = 0.22 (−0.04;0.46)	–
				Adolescents: sedentary(Q1) – sedentary(Q2)	ICC (95 % CI) = 0.57 (0.34;0.73)	–
				Adults: sedentary(Q1) – sedentary(Q2)	ICC (95 % CI) = 0.60 (0.40;0.74)	–
Adults	Craig (2003)[34]	3 - 7 days	IPAQ	Long form: total PA(Q1) – total PA(Q2)	Pooled Spearman r (95 % CI) = 0.81 (0.79;0.82), range: 0.46 - 0.96	–
				Short form: total PA(Q1) – total PA(Q2)	Pooled Spearman r (95 % CI) = 0.76 (0.73;0.77), range: 0.32 - 0.88	–
Adults	Fjeldsoe (2009)[35]	7 days	AWAS	Total activity(Q1) – total activity(Q2)	ICC (95 % CI) = 0.73 (0.51;0.86)	–
				HEPA(Q1) – HEPA(Q2)	ICC (95 % CI) = 0.80 (0.65;0.89)	–
				Sitting(Q1) – sitting(Q2)	ICC (95 % CI) = 0.42 (0.13;0.64)	–
Adults	Friedenreich (2006)[36]	9 weeks (average)	PYTPAQ	Total MET-hr/week(Q1) – total MET-hr/week(Q2)	ICC (95 % CI) = 0.66 (0.56;0.74), Spearman r = 0.64 (P < 0.0001)	–
Adults	Kurtze (2007)[37]	7 days	HUNT2	Hard activity(Q1) – hard activity(Q2)	Spearman r = 0.17 (P < 0.01)	κ = 0.41 (0.29;0.54)
				Occupational activity(Q1) – occupational activity(Q2)	Spearman r = 0.85 (P < 0.01)	κ = 0.80 (0.71;0.89)
				Light activity(Q1) – light activity(Q2)	Spearman r = 0.17	κ = 0.20 (0.04;0.35)
Adults	Kurtze (2008)[38]	7 days	HUNT1	Frequency(Q1) – frequency(Q2)	Spearman r = 0.87 (P < 0.01)	κ = 0.80
				Intensity(Q1) – intensity(Q2)	Spearman r = 0.87 (P < 0.01)	κ = 0.82
				Duration(Q1) – duration(Q2)	Spearman r = 0.76 (P < 0.01)	κ = 0.69
Adults	Lowther (1999)[39]	2 days	SPAQ	Total min(Q1) – total min(Q2)	Pearson r = 0.998 (P < 0.01), repeatability coefficient R = 53 min.	MD (95 % LoA) = 3.09 ± 26.5 min
Adults	Mäder (2006)[40]	14 - 21 days	SHS97	Sweat episodes(Q1) – sweat episodes(Q2)	Spearman r = 0.63 (P < 0.05)	–
			HEPA99	Active/inactive(Q1) – active/inactive(Q2)	–	κ = 0.46 (P < 005)
			IPAQ	Total MET-min/week(Q1) – total MET-min/week(Q2)	Spearman r = 0.54 (P < 0.05)	–
				Sitting(Q1) – sitting(Q2)	Spearman r = 0.60 (P < 0.05)	–
			OIMQ	Total MET-min/week(Q1) – total MET-min/week(Q2)	Spearman r = 0.68 (P < 0.05)	–
Adults	Meriwether (2006)[41]	7 days	PAAT	Total min(Q1) – total min(Q2)	Spearman r = 0.618 (P < 0.001)	–
Adults	Reis (2005)[42]	2 weeks	OPAQ	Total activity(Q1) – total activity(Q2)	ICC (95 % CI) = 0.76 (0.59;0.86)	–
				Sedentary(Q1) – sedentary(Q2)	ICC (95 % CI) = 0.78 (0.62;0.87)	–
Adults	Rosenberg (2010)[43]	2 weeks	SBQ	Weekday: total score(Q1) – total score(Q2)	ICC (95 % CI) = 0.85 (0.75;0.91), Spearman r (95 % CI) = 0.79 (0.65;0.88)	–
				Weekend day: total score(Q1) – total score(Q2)	ICC (95 % CI) = 0.77 (0.63;0.86), Spearman r (95 % CI) = 0.74 (0.58;0.85)	–
Adults	Sobngwi (2001)[44]	10 - 15 days	SSAAQ	Total min(Q1) – total min(Q2)	Spearman r = 0.95 (P < 0.001)	–
Adults	Timperio (2003)[45]	3 days	1WPAR	Men: duration(Q1) – duration(Q2)	ICC (95 % CI) = 0.45 (0.20;0.64), P < 0.001	–
				Women: duration(Q1) – duration(Q2)	ICC (95 % CI) = 0.80 (0.69;0.87), P < 0.001	–
				Men: sufficient PA(Q1) – sufficient PA(Q2)	–	κ = 0.64 (P < 0.001)
				Women: sufficient PA(Q1) – sufficient PA(Q2)	–	κ = 0.55 (P < 0.001)
Adults	Wareham (2002)[46]	3 months	EPAQ2	Men: total MET-hr/week(Q1) – total MET-hr/week(Q2)	Pearson r = 0.74 (P < 0.05)	κ = 0.64
				Women: total MET-hr/week(Q1) – total MET-hr/week(Q2)	Pearson r = 0.72 (P < 0.05)	κ = 0.70
				Men: TV time(Q1) – TV time(Q2)	Pearson r = 0.75 (P < 0.05)	κ = 0.71
				Women: TV time(Q1) – TV time(Q2)	Pearson r = 0.78 (P < 0.05)	κ = 0.74
Adults	Wareham (2003)[47]	18 - 21 months	EPAQ	Physical activity index(Q1) – physical activity index(Q2)	–	κ = 0.60 (P < 0.0001)
Adults	Yore (2007)[48]	1 - 5 days	BRFSS PAQ	VPA(Q1) – VPA(Q2)	–	κ (95 % CI) = 0.86 (0.72;0.99)
				MPA(Q1) – MPA(Q2)	–	κ (95 % CI) = 0.53 (0.31;0.75)
				Recommended PA(Q1) – recommended PA(Q2)	–	κ (95 % CI) = 0.84 (0.69;0.99)
				Walking(Q1) – walking(Q2)	–	κ (95 % CI) = 0.56 (0.34;0.77)
				Strengthening PA(Q1) – strengthening PA(Q2)	–	κ (95 % CI) = 0.92 (0.81;1.00)
		10 - 19 days	BRFSS PAQ	VPA(Q1) – VPA(Q3)	–	κ (95 % CI) = 0.80 (0.65;0.95)
				MPA(Q1) – MPA(Q3)	–	κ (95 % CI) = 0.35 (0.11;0.59)
				Recommended PA(Q1) – recommended PA(Q3)	–	κ (95 % CI) = 0.67 (0.46;0.88)
				Walking(Q1) – walking(Q3)	–	κ (95 % CI) = 0.34 (0.10;0.57)
				Strengthening PA(Q1) – strengthening PA(Q3)	–	κ (95 % CI) = 0.85 (0.71;0.99)
Elderly	Yasunaga (2007)[49]	1 month	PAQ-EJ	PAQ-EJ score(Q1) – PAQ-EJ score(Q2)	Pearson r = 0.70 (P < 0.05)	–
					*Median ICC = 0.76 (youth: 0.69, adults: 0.765, elderly: –)*	
					*Median Spearman r = 0.74 (youth: 0.71, adults: 0.75, elderly: –)*	
					*Median Pearson r = 0.76 (youth: 0.80, adults: 0.74, elderly: 0.70)*	
						*Median κ = 0.64 (youth: 0.53, adults: 0.655, elderly: –)*

Q1 =first completed questionnaire, Q2 = second completed questionnaire, Q3 = third completed questionnaire, r = correlation coefficient (rho), ICC = Intraclass Correlation Coefficient, CI = Confidence Interval (lower;upper), %CV = coefficient of variation (within subjects standard deviation of typical error) as a percentage of the mean score, κ = kappa (i.e. Cohen weighted kappa unless specified otherwise), LoA = Limits of Agreement, MD = Mean Difference, – = not stated.

**NB:** No calculation of weighted kappa is specified in the papers. Usually the kappa statistic is used for categorical responses and weighted kappa for ordinal responses. Interpretation of values of kappa and weighted kappa were usually based on the classification system developed by Landis and Koch (1977), where <0.10 indicated poor agreement, 0.10-0.20 slight agreement, 0.21-0.40 fair agreement, 0.41-0.60 moderate agreement, 0.61-0.80 substantial agreement, 0.81-1.00 almost perfect agreement.

Ainsworth (2000): 3 point summary index = 3 domains: sports/exercise, occupation, active living habits. 4 point summary index = all 4 domains: sports/exercise, occupation, active living habits, housework/caregiving.

Chinapaw (2009): AQuAA score: all activities above 2 MET in MET-min/week.

Craig (2003): Pooled Spearman = pooled results from data of 22 studies examining the IPAQ long form and 23 studies examining the short form.

Dwyer (2011): Levels 1–2 = stationary, level 3 = moving slowly, level 4 = moving at a medium or moderate pace, level 5 = moving at a fast pace.

Economos (2010): Moderate-high METs = 3–6 METs. High METs = ≥6 METs. WBF score = weight-bearing factor score, calculated by adding the weight-bearing factor of the reported weight-bearing activities.

Fjeldsoe (2009): HEPA = Health Enhancing Physical Activity: brisk walking and moderate- and vigorous activities from the planned activity and transport domains.

Kurtze (2007): Light activity = no sweating or being out of breath. Hard activity = sweating/out of breath.

Lowther (1999): Total min = total minutes measured in the overlapping 4 days of both questionnaires. Repeatability coefficient (twice the standard deviation of the differences) means that 95 % of the differences in SPAQ from one measurement to the next (under similar conditions) would be between zero plus or minus 53 minutes.

Mäder (2006): IPAQ - Total MET-min/week = MET-min/week for total activity excluding sitting. OIMQ - Total MET-min/week = MET-min/week for total activity, i.e. moderate and vigorous activities.

Philippaerts (2006): Total hrs/week = Total hours per week spent in transport and sports participation, excluding sedentary activities. Total EE = Total EE spent in transport and sports participation, excluding sedentary activities.

Reis (2005): Sedentary = sitting or standing activities.

Ridley (2001): CDPAQ-HC = hard copy of CDPAQ.

Rosenberg (2010): Total score = all sedentary behaviors in hours per day for each item were summed separately for weekday and weekend days.

Telford (2004): Reliability results for frequency/duration of overall total PA for 5 to 6 or 10 to 12 year old children in parental proxy-reports or self-administered questionnaires.

Timperio (2003): Duration = duration of total physical activity. Sufficient PA was calculated as 150 minutes of combined walking, moderate- and vigorous-intensity physical activity, with reported duration of vigorous-intensity physical activity weighted by two.

Treuth (2003): GAQ score = MET weighted mean score of 28 activities. Activitygram score = average intensity/min. Other sedentary = sedentary activities excluding TV watching.

Treuth (2005): Fels PAQ score = total activity score; MET weighted sum of sport, leisure, work index.

Wareham (2003): Physical activity index is a four-category index of inactive, moderately inactive, moderately active, active. TV time = hours per week watching television and videos.

Wong (2006): Combined activity = combined score of the SHAPES derived variables which contains the variables: VPA, MPA, MVPA, screen time, PAL and BMI.

Yasunaga (2007): PAQ-EJ score (MET-hr/week) = number of days*time*intensity weight.

Yore (2007): MPA ≥ 30 min/day on 5 days/week. VPA ≥ 20 min/day on 3 days/week. Recommended PA, i.e. ≥ subjects who met the criteria for moderate or vigorous PA. Walking ≥ 30 min/day. Strengthening PA = any muscle-strengthening activity on ≥ 2 days/week. Kappa's are reported for the subsamples who met the criteria for the physical activity intensities.

Reliability was usually reported as ICC (n = 13), Pearson/Spearman correlation (n = 6), kappa statistic (n = 3) or a combination of these statistics (n = 9). Higher reliability coefficients were more often seen in association with shorter periods between test and retest. Poor correlation (ICC or r <0.4) was found only in subcategories of a few PAQs. Median correlations from reported data for recall of sedentary behaviours across all PAQs were acceptable: ICC = 0.68, Spearman r = 0.60, Pearson r = 0.475, kappa = 0.66.

### Youth

Median reliability correlations for the youth were as follows: ICC = 0.69, Spearman r = 0.71, Pearson r = 0.80, kappa = 0.53. The Activitygram (ICC = 0.24) [26] and the self-reported CLASS questionnaire (frequency: ICC = 0.36, duration ICC = 0.24) [25] showed fairly low reliability correlations, whereas the MARCA (ICC = 0.93) [52] and both computer and paper versions of the CDPAQ (ICC = 0.91–0.98) [23] demonstrated high reliability.

### Adults

Median reliability correlations for adults were as follows: ICC = 0.765, Spearman r = 0.75, Pearson r = 0.74, kappa = 0.655. Reliability was poor for the AQuAA score for adults (ICC = 0.22) [53]. Similarly, reliability coefficients were poor for the HUNT2 [37] components of light (r = 0.17, κ = 0.20) and hard activity (r = 0.17, κ = 0.41). The primary version of this questionnaire (HUNT1), which was designed a decade earlier, however demonstrated high reliability (r = 0.76–0.87, κ = 0.69–0.82) [54]. The majority of the questionnaires showed acceptable to good reliability: KPAS (ICC = 0.82–0.83) [30], RPAQ (ICC = 0.76) [31], PPAQ (ICC = 0.78) [32], IPAQ short (r = 0.76) and long version (r = 0.81) [34], AWAS (ICC = 0.73–0.80) [35], FPACQ (ICC = 0.68–0.80) [22], OPAQ (ICC = 0.78) [42], SBQ (ICC = 0.77-0.85, r = 0.74-0.79) [43], SPAQ (r = 0.998) [39] and SSAAQ (r = 0.95) [44].

### Elderly

Median Pearson reliability correlation for the elderly was r = 0.70. The PAQ-EJ was the only new PAQ designed for (Japanese) elderly that reported reliability results and has acceptable recall properties (r = 0.70) [49].

### Validity

All validity results for new PAQs are listed in Table 4.

**Table 4:** Validity results of new PAQs

**Age Group**	**Reference**	**Criterion method**	**Duration of validation**	**PAQ**	**Variables tested**	**Criterion intensity thresholds**	**Validity results**	
							***Correlation coefficients***	***Agreement***
Youth	Dwyer (2011)[19]	Acc (ActiGraph)	4 - 5 days	Pre-PAQ	Level 5 min/day(Q) – VPA min/day(Acc)	>5016 counts/min	Pearson r = 0.17	MD (95 % LoA) = 1.9 ± 39.4 min/day
					Level 4 min/day(Q) – MPA min/day(Acc)	3560-5016 counts/min	Pearson r = 0.13	MD (95 % LoA) = 48.2 ± 73.1 min/day
					Level 3 min/day(Q) – LPA min/day(Acc)	1592-3560 counts/min	Pearson r = −0.07	MD (95 % LoA) = −4.8 ± 100.7 min/day
					Levels 1–2 min/day(Q) – sedentary min/day(Acc)	<1592 counts/min	Pearson r = 0.19	MD (95 % LoA) = −235.4 ± 147.7 min/day
Youth	Economos (2010)[20]	Acc (ActiGraph)	2 days	BONES PAS	High METs(Q) – total counts/min(Acc)	–	Spearman r (95 % CI) = 0.25 (−0.07;0.52)	–
					High METs(Q) – VPA(Acc)	6-9 METs, 1952–5724 counts/min	Spearman r (95 % CI) = 0.23 (−0.09;0.51)	–
					Moderate-high METs(Q) – total counts/min(Acc)	–	Spearman r (95 % CI) = 0.27 (−0.05;0.54)	–
Youth	Martinez-Gomez (2010)[21]	Acc (ActiGraph)	1 day	RPAR	Total MET-min(Q) – total counts(Acc)	–	Pearson r = 0.42 (P = 0.021)	κ = 0.16
					MVPA min(Q) – MVPA counts(Acc)	≥2000 counts/min	Pearson r = 0.52 (P < 0.001)	MD (95 % LoA) = 2.15 ± 7.19 min
		Acc (Biotrainer)	1 day		Total MET-min(Q) – total counts(Acc)	–	Pearson r = 0.40 (P = 0.025)	κ = 0.39
					Total MET-min(Q) – total counts/mov(Acc)	–	Pearson r = 0.54 (P = 0.004)	κ = 0.16
Youth	Philippaerts (2006)[22]	Acc (ActiGraph)	7 days	FPACQ	Total hr/week(Q) – total counts(Acc)	–	Pearson r = 0.56 (P < 0.01)	–
					Total hr/week(Q) – mean counts/min(Acc)	–	Pearson r = 0.43 (P < 0.05)	–
					TEE(Q) – total counts(Acc)	–	Pearson r = 0.58 (P < 0.01)	–
					TEE(Q) – mean counts/min(Acc)	–	Pearson r = 0.49 (P < 0.05)	–
					Inactivity(Q) – total counts(Acc)	–	Pearson r = −0.13	–
					Inactivity(Q) – mean counts/min(Acc)	–	Pearson r = −0.06	–
Youth	Ridley (2001)[23]	Acc (Caltrac)	2x 1 day	CDPAQ	Total METs(Q) – total counts(Acc)	–	Pearson r = 0.41 (P < 0.05)	–
					Total compendium METs(Q) – total counts(Acc)	–	Pearson r = 0.54 (P < 0.05)	–
					Total mins(Q) – total counts(Acc)	–	Pearson r = 0.41 (P < 0.05)	–
		HR (Polar)	2x 1 day		MVPA mins(Q) – MVPA mins(HR)	≥145 bpm	Pearson r = 0.66 (P = 0.01)	–
		Acc (Caltrac)	2x 1 day	CDPAQ-HC	Total METs(Q) – total counts(Acc)	–	Pearson r = 0.25 (P < 0.05)	–
					Total compendium METs(Q) – total counts(Acc)	–	Pearson r = 0.22 (P < 0.05)	–
					Total mins(Q) – total counts(Acc)	–	Pearson r = 0.33 (P < 0.05)	–
		HR (Polar)	2x 1 day		MVPA mins(Q) – MVPA mins(HR)	≥145 bpm	Pearson r = 0.48 (P = 0.05)	–
Youth	Ridley (2006)[24]	Acc (ActiGraph)	1 day	MARCA	PAL(Q) – total counts(Acc)	–	Spearman r = 0.45 (P < 0.01)	–
Youth	Telford (2004)[25]	Acc (ActiGraph)	8 days	CLASS-parental report	5-6 yrs: total min/day(Q) – total min/day(Acc)	–	Spearman r = −0.04	MD (95 % LoA) = −140.7 (−164.9;-116.6) min/day
					10-12 yrs: total min/day(Q) – total min/day(Acc)	–	Spearman r = 0.09	MD (95 % LoA) = 11.2 (−6.9;29.4) min/day
					5-6 yrs: total min/day(Q) – total raw counts/day(Acc)	–	Spearman r = 0.05	–
					10-12 yrs: total min/day(Q) – total raw counts/day(Acc)	–	Spearman r = 0.11	–
				CLASS-self	10-12 yrs: total min/day(Q) – total min/day(Acc)	–	Spearman r = −0.04	MD (95 % LoA) = 1.5 (−17.2;20.3) min/day
					10-12 yrs: total min/day(Q) – total raw counts/day(Acc)	–	Spearman r = 0.06	–
Youth	Treuth (2003)[26]	Acc (ActiGraph)	4 days	GAQ	Yesterday: GAQ score(Q) – mean counts/min(Acc)	–	Pearson r = 0.27 (P < 0.05)	–
					Usual: GAQ score(Q) – mean counts/min(Acc)	–	Pearson r = 0.29 (P < 0.05)	–
					Yesterday: TV watching(Q) – mean counts/min(Acc)	–	Pearson r = −0.145 (P = 0.24)	–
					Usual: TV watching(Q) – mean counts/min(Acc)	–	Pearson r = −0.004 (P = 0.98)	–
					Yesterday: other sedentary(Q) – mean counts/min(Acc)	–	Pearson r = 0.0227 (P = 0.85)	–
					Usual: other sedentary(Q) – mean counts/min(Acc)	–	Pearson r = −0.0916 (P = 0.46)	–
				Activitygram	Activitygram score(Q) – mean counts/min(Acc)	–	Pearson r = 0.37 (P < 0.002)	–
Youth	Treuth (2005)[27]	Acc (Actiwatch)	6 days	Fels PAQ	Elementary: Fels PAQ score(Q) – mean counts/min(Acc)	–	Spearman r = 0.34 (P = 0.004)	–
					Middle: Fels PAQ score(Q) – mean counts/min(Acc)	–	Spearman r = 0.11 (P = 0.31)	–
					High: Fels PAQ score(Q) – mean counts/min(Acc)	–	Spearman r = 0.21 (P = 0.006)	–
Youth	Welk (2007)[28]	Acc (ActiGraph)	7 days	YMCLS	Weekly PA bouts(Q) – weekly PA bouts(Acc)	–	r = 0.24 (P < 0.05)	MD (95 % LoA) = −8.4 ± 28.4 min
					Previous day: total MVPA mins(Q) – total MVPA mins(Acc)	3-6 METs	r = 0.53 (P < 0.05)	MD (95 % LoA) = 14.5 ± 173.9 min
Youth	Wong (2006)[29]	Acc (ActiGraph)	7 - 9 days	SHAPES	VPA min/day(Q) – VPA min/day(Acc)	≥8200 counts/min	Spearman r = 0.25 (P = 0.07)	–
					MVPA min/day(Q) – MVPA min/day(Acc)	≥3200 counts/min	Spearman r = 0.44 (P < 0.01)	–
					MPA min/day(Q) – MPA min/day(Acc)	3200-8199 counts/min	Spearman r = 0.31 (P = 0.02)	–
Adults	Ainsworth (2000)[30]	Acc (Caltrac)	2x 7 days	KPAS	3 point summary index(Q) – MET-min/day(Acc)	–	Spearman r = 0.53 (P < 0.01)	–
					4 point summary index(Q) – MET-min/day(Acc)	–	Spearman r = 0.49 (P < 0.01)	–
Adults	Besson (2010)[31]	DLW	14 days	RPAQ	TEE(Q) – TEE(DLW)	–	Spearman r = 0.67 (P < 0.0001)	MD (95 % LoA) = −3451.9 ± 2025.1 kJ/day (P < 0.05)
					PAEE(Q) – PAEE(DLW)	–	Spearman r = 0.39 (P = 0.0004)	MD (95 % LoA) = −12.9 ± 23.9 kJ/day (P < 0.05)
		Acc + HR (Actiheart)	11 days		VPA(Q) – VPA(Acc + HR)	>6 METs	Spearman r = 0.70 (P < 0.0001)	MD (95 % LoA) = 0.2 ± 0.4 h/day
					MPA(Q) – MPA(Acc + HR)	3.6-6 METs	–	MD (95 % LoA) = −0.8 ± 1.0 h/day
					Light PA(Q) – light PA(Acc + HR)	2-3.5 METs	–	MD (95 % LoA) = −0.1 ± 2.4 h/day
					Sedentary time(Q) – sedentary time(Acc + HR)	<2 METs	Spearman r = 0.27 (P = 0.06)	MD (95 % LoA) = 0.7 ± 2.8 h/day
Adults	Chasan-Taber (2004)[32]	Acc (ActiGraph)	7 days	PPAQ	Total activity(Q) – Swartz cut point min/day(Acc)	≥3 METs, ≥574 counts/min	Spearman r = 0.32	–
					Total activity(Q) – Hendelman cut point min/day(Acc)	≥3 METs, ≥191 counts/min	Spearman r = 0.43	–
					Total activity(Q) – Freedson cut point min/day(Acc)	≥3 METs, ≥1952 counts/min	Spearman r = 0.08	–
					Total activity(Q) – mean counts/min(Acc)	–	Spearman r = 0.27	–
					Sedentary(Q) – Swartz cut point min/day(Acc)	<1.5 METs	Spearman r = −0.17	–
					Sedentary(Q) – Hendelman cut point min/day(Acc)	<1.5 METs	Spearman r = −0.34	–
					Sedentary(Q) – Freedson cut point min/day(Acc)	<1.5 METs	Spearman r = 0.12	–
					Sedentary(Q) – mean counts/min(Acc)	–	Spearman r = −0.10	–
Adults	Chinapaw (2009)[33]	Acc (ActiGraph)	14 days	AQuAA	Adolescents: AQuAA score(Q) – counts/min(Acc)	≥ 2 METs, ≥699 counts/min	Spearman r = 0.13	–
					Adults: AQuAA score(Q) – counts/min(Acc)	≥ 2 METs, ≥699 counts/min	Spearman r = −0.16	–
					Adolescents: sedentary(Q) – counts/min(Acc)	< 2 METs, <699 counts/min	Spearman r = 0.23	–
					Adults: sedentary(Q) – counts/min(Acc)	< 2 METs, <699 counts/min	Spearman r = 0.15	–
Adults	Craig (2003)[34]	Acc (ActiGraph)	7 days	IPAQ	Long form: total PA(Q) – total counts(Acc)	–	Pooled Spearman r (95 % CI) = 0.33 (0.26;0.39), range: -0.27 - 0.61	–
					Short form: total PA(Q) – total counts(Acc)	–	Pooled Spearman r (95 % CI) = 0.30 (0.23;0.36), range: -0.12 - 0.57	–
Adults	Fjeldsoe (2009)[35]	Acc (ActiGraph)	7 days	AWAS	Total activity(Q) – total activity(Acc)	≥100 counts/min	Spearman r = 0.13 (P = 0.24)	–
					HEPA(Q) – Freedson cut point min/week(Acc)	–	Spearman r = 0.28 (P = 0.01)	–
					HEPA(Q) – Swartz cut point min/week(Acc)	–	Spearman r = 0.06 (P = 0.64)	–
					Sitting(Q) – sitting(Acc)	<100 counts/min	Spearman r = 0.32 (P = 0.006)	–
Adults	Friedenreich (2006)[36]	Acc (ActiGraph)	4x 7 days	PYTPAQ	Total MET-hr/week(Q) – total MET-hr/week(Acc)	–	Spearman r = 0.26 (P < 0.05), ICC (95 % CI) = 0.18 (0.03;0.32)	–
Adults	Kurtze (2007)[37]	Acc (ActiReg)	7 days	HUNT2	Hard activity(Q) – EE(Acc)	–	Spearman r = 0.11	–
					Hard activity(Q) – PAL(Acc)	–	Spearman r = 0.16	–
					Light activity(Q) – EE(Acc)	–	Spearman r = 0.21 (P < 0.05)	–
					Light activity(Q) – PAL(Acc)	–	Spearman r = 0.08	–
					Occupational activity(Q) – EE(Acc)	–	Spearman r = 0.39 (P < 0.01)	–
					Occupational activity(Q) – PAL(Acc)	–	Spearman r = 0.38 (P < 0.01)	–
Adults	Kurtze (2008)[38]	Acc (ActiReg)	7 days	HUNT1	Summary index(Q) – EE(Acc)	–	Spearman r = 0.03	–
					Summary index(Q) – PAL(Acc)	–	Spearman r = 0.07	–
					Summary index(Q) – MET-min/day(Acc)	–	Spearman r = 0.07	–
Adults	Lowther (1999)[39]	Acc (Caltrac)	4 days	SPAQ	Total mins(Q) – total kcal(Acc)	–	r = 0.1294, corrected for confounding: r = 0.52 (P < 0.05)	–
Adults	Mäder (2006)[40]	Acc (ActiGraph)	7 days	SHS97	Sweat episodes/week(Q) – total counts/min(Acc)	–	Spearman r = 0.23	–
				HEPA99	–	–	–	–
				IPAQ	Total MET-min/week(Q) – total counts/min(Acc)	–	Spearman r = 0.39 (P < 0.05)	–
					Sitting(Q) – sitting(Acc)	<100 counts/min	Spearman r = 0.22	–
				OIMQ	Total MET-min/week(Q) – total counts/min(Acc)	–	Spearman r = 0.44 (P < 0.05)	–
Adults	Meriwether (2006)[41]	Acc (MTI)	14 days	PAAT	VPA min/week(Q) – VPA min/week(Acc)	≥5 METs, ≥5725 counts/min	Spearman r = 0.380 (P < 0.01)	–
					MVPA min/week(Q) – MVPA min/week(Acc)	≥5 METs, ≥1952 counts/min	Spearman r = 0.392 (P < 0.01)	–
					MPA min/week(Q) – MPA min/week(Acc)	3-4.9 METs, 1952–5724 counts/min	Spearman r = 0.392 (P < 0.01)	–
Adults	Reis (2005)[42]	Acc (ActiGraph)	7 days	OPAQ	Total hr/week(Q) – VPA(Acc)	≥5725 counts/min	Spearman r = −0.02	–
					Total hr/week(Q) – MPA(Acc)	1952-5724 counts/min	Spearman r = 0.12	–
					Total hr/week(Q) – light activity(Acc)	<1952 counts/min	Spearman r = 0.22	–
					Sedentary(Q) – light activity(Acc)	<1952 counts/min	Spearman r = −0.20	–
Adults	Rosenberg (2010)[43]	Acc (ActiGraph)	7 days	SBQ	Female: total sedentary hr/week(Q) – total sedentary counts(Acc)	<100 counts/min	Partial r = 0.10 (P = 0.07)	–
					Male: total sedentary hr/week(Q) – total sedentary counts(Acc)	<100 counts/min	Partial r = −0.01 (P = 0.81)	–
Adults	Sobngwi (2001)[44]	Acc (Caltrac)	1 day	SSAAQ	Female: total METs(Q) – total METs(Acc)	–	r = 0.74 (P < 0.01)	–
					Male: total METs(Q) – total METs(Acc)	–	r = 0.60 (P < 0.01)	–
		HR (Polar)	1 day		Urban female: total METs(Q) – total activity(HR)	–	r = 0.63 (P < 0.01)	–
					Rural female: total METs(Q) – total activity(HR)	–	r = 0.41 (P < 0.05)	–
					Urban male: total METs(Q) – total activity(HR)	–	r = 0.54 (P < 0.05)	–
					Rural male: total METs(Q) – total activity(HR)	–	r = 0.59 (P < 0.01)	–
Adults	Timperio (2003)[45]	Acc (ActiGraph)	7 days	1WPAR	Men: total min/day(Q) – total min/day(Acc)	≥3 METs, ≥1952 counts/min	Spearman r = 0.29 (P < 0.05)	–
					Women: total min/day(Q) – total min/day(Acc)	≥3 METs, ≥1952 counts/min	Spearman r = 0.25 (P < 0.05)	–
Adults	Wareham (2002)[46]	HR (Polar)	4x 4 days	EPAQ2	Total MET-hr/week(Q) – EE(HR)	–	Pearson partial r = 0.28 (P < 0.001)	–
					TV time(Q) – EE(HR)	–	Pearson partial r = −0.07	–
Adults	Wareham (2003)[47]	HR (Polar)	4x 4 days	EPAQ-s	Physical activity index(Q) – DayPAR(HR)	–	P for trend = 0.003	–
					Total hr/week(Q) – DayPAR(HR)	–	r = 0.04 (P = 0.59)	–
Adults	Yore (2007)[48]	Acc (ActiGraph)	7 days	BRFSS PAQ	VPA min/week(Q1) – VPA min/week(Acc)	≥5999 counts/min	Pearson r = 0.52	–
					VPA min/week(Q2) – VPA min/week(Acc)	≥5999 counts/min	Pearson r = 0.54	–
					VPA min/week(Q3) – VPA min/week(Acc)	≥5999 counts/min	Pearson r = 0.63	–
					MPA min/week(Q1) – MPA min/week(Acc)	2020-5998 counts/min	Pearson r = 0.27	–
					MPA min/week(Q2) – MPA min/week(Acc)	2020-5998 counts/min	Pearson r = 0.20	–
					MPA min/week(Q3) – MPA min/week(Acc)	2020-5998 counts/min	Pearson r = 0.16	–
Elderly	Yasunaga (2007)[49]	Acc (Kenz Lifecorder)	1 month	PAQ-EJ	PAQ-EJ score(Q) – MET-min/day(Acc)	–	Spearman r = 0.41 (P < 0.05)	–
							*Median Spearman r = 0.25 (youth: 0.22, adults: 0.27, elderly: 0.41)*	
							*Median Pearson r = 0.41 (youth: 0.41, adults: 0.28, elderly: –)*	

Q1 = first completed questionnaire, Q2 = second completed questionnaire, Q3 = third completed questionnaire, r = correlation coefficient (rho), CI = Confidence Interval (lower;upper), κ = kappa (i.e. Cohen weighted kappa unless specified otherwise), LoA = Limits of Agreement, MD = Mean Difference, – = not stated.

Acc = Accelerometry [NB: ActiGraph (Model 7164) is successor of preceding accelerometer by MTI, formerly CSA]. Accelerometer names as used in the respective papers.

Ainsworth (2000): 3 point summary index = 3 domains: sports/exercise, occupation, active living habits. 4 point summary index = all 4 domains: sports/exercise, occupation, active living habits, housework/caregiving.

Craig (2003): Pooled Spearman = pooled results from data of 22 studies examining the IPAQ long form and 23 studies examining the short form.

Dwyer (2011): Levels 1–2 = stationary, level 3 = moving slowly, level 4 = moving at a medium or moderate pace, level 5 = moving at a fast pace.

Economos (2010): Moderate-high METs = 3–6 METs. High METs = ≥6 METs.

Fjeldsoe (2009): Total activity includes light-, moderate-, and vigorous-intensity activities. HEPA = Health Enhancing Physical Activity: brisk walking and moderate- and vigorous activities from the planned activity and transport domains.

Kurtze (2007): EE = Energy Expenditure in MJ/day. PAL = total EE divided by basal metabolic rate (BMR). Light activity = no sweating or being out of breath. Hard activity = sweating/out of breath.

Kurtze (2008): EE = Energy Expenditure in MJ/day. PAL = total EE divided by basal metabolic rate (BMR).

Lowther (1999): Initial r = 0.1294, but after correction for less reliable high data (occupational walking data, extreme data for 4 participants) the correlation improved to 0.52.

Mäder (2006): IPAQ - Total MET-min/week = MET-min/week for total activity excluding sitting. OIMQ - Total MET-min/week = MET-min/week for total activity, i.e. moderate and vigorous activities.

Martinez-Gomez (2010): Counts/mov = counts adjusted by movement time over the recess time. MD = mean difference between the mean times spent at MVPA by the two instruments. Kappa = agreement between the two instruments among tertiles of total PA.

Reis (2005): ActiGraph only worn during occupational hours. Sedentary = sitting or standing activities.

Ridley (2001): CDPAQ-HC = hard copy of CDPAQ. MVPA = Moderate-to-Vigorous Physical Activity. Total compendium METs = compendium values to derive total METs due to reported problems associated with children's perception of intensity (Compendium of physical activities: classification of energy costs of human physical activities. Ainsworth BE, Haskell WL, Leon AS, Jacobs DR Jr, Montoye HJ, Sallis JF, Paffenbarger RS Jr. Med Sci Sports Exerc. 1993 Jan;25(1):71–80).

Rosenberg (2010): Partial r = partial correlation, adjusted for age, marital status, white or nonwhite ethnicity, number of children, and highest level of education.

Sobngwi (2001): Total activity by Heart Rate monitoring is defined as variability in heart rate measured as area under the minute-to-minute heart rate curve and above individual resting heart rate.

Telford (2004): Validity results for total PA minutes for 5 to 6 or 10 to 12 year old children in parental proxy-reports or self-administered questionnaires.

Timperio (2003): Total activity in min/day is specified as ≥3 METs.

Treuth (2003): GAQ score = MET weighted mean score of 18 more reliable, and more frequently performed, activities. Activitygram score = average intensity/min over 3 day period. Other sedentary = sedentary activities excluding TV watching. The scores are an average of the two days administrations.

Treuth (2005): Fels PAQ score = mean Fels PAQ score (total activity) of both administrations of the PAQ. Counts/min = mean counts/min. Elementary = elementary school. Middle = middle school. High = high school.

Wareham (2002): Subject wore the HR monitor 4x four days across one year. EE = Energy Expenditure in kJ/hr. TV time = hours per week watching television and videos. Partial correlation coefficient is adjusted for age and sex.

Wareham (2003): Subject wore the HR monitor 4x four days across one year. Physical activity index = combined index for the four-level classification of self-reported occupational activity and four-level categorisation of time spent in cycling and other physical exercise. DayPAR = Physical Activity Ratio calculated as the ratio of daytime energy expenditure to resting energy expenditure. P for trend = P for positive trend of the association between DayPAR (measured by calibrated HR data) over four categories of physical activity (i.e. inactive, moderately inactive, moderately active, active) estimated from the EPAQ.

Welk (2007): PA bouts = number of sessions of physical activity performed during the week. Total MVPA mins = total minutes in moderate to vigorous physical activity performed during the previous day. Cut point used is Freedson age-based cut point, calculated as METs = 2.757 + (0.0015*counts per minute) - (0.0896*age[yr]) - (0.000038*counts per minute*age[yr]). Correlation = group-level correlation. No correlation coefficient specified.

Yasunaga (2007): PAQ-EJ score = MET score in MET-hr/week, calculated as number of days*time*intensity weight.

Accelerometry and in particular the ActiGraph accelerometer was the most commonly used criterion method (n = 19), followed by the Caltrac accelerometer (n = 4) and the Polar heart rate monitor (n = 4). DLW was used in one study, where absolute validity was moderate to high for PAEE (r = 0.39) and TEE (r = 0.67) [31]. In general, validity coefficients were considerably lower than reliability coefficients. Median correlations across all PAQs between reported sedentary behaviours and calculated inactivity from objective measures were low: Spearman r = 0.12.

### Youth

Median validity correlations for the youth were as follows: Spearman r = 0.22, Pearson r = 0.41. CLASS self- and parental reported physical activity (r = −0.04–0.11) [25] was among the least valid questionnaires for children, although several other PAQs also showed low correlations with objective measures: Pre-PAQ (r = −0.07–0.17) [19], BONES PAS (r = 0.23–0.27) [20], GAQ (r = 0.27–0.29) [26], Fels PAQ (0.11–0.34) [27]. None of the newly developed PAQs for children demonstrated high validity.

### Adults

Median validity correlations for adults were as follows: Spearman r = 0.27, Pearson r = 0.28. Highest validity in adults was demonstrated for the SSAAQ when tested against the Caltrac accelerometer (r = 0.60-0.74) [44]. Low validity correlations for total activity or for all subcategories were observed for the HUNT1 (r = 0.03–0.07) [54], and the short EPIC PAQ (r = 0.04), although the main outcome, a 4 category physical activity index, derived from this instrument was significantly associated with objectively measured physical activity energy expenditure (p for trend = 0.003) [47]. A follow-up study in 1941 adults from 10 European countries suggested moderate validity (r = 0.33) of this instrument using physical activity energy expenditure from combined heart rate and movement sensing as the criterion [51].

Rosenberg et al. assessed the validity of sedentary behaviour only, and demonstrated low correlations (partial r = −0.01–0.10) with objectively measured sedentary time (<100 counts/min) by the ActiGraph accelerometer [43].

### Elderly

Median Spearman validity correlation for the elderly was r = 0.41. The PAQ-EJ was tested by correlating a total score with MET-min/day calculated from the Kenz Lifecorder accelerometer-based pedometer (r = 0.41) [49].

### Existing PAQs

New validity and reliability results for existing PAQs were reported in 35 studies, and 30 studies reported new results on validity only (Table 5). One study is classified as a study testing an existing PAQs, but also reports both validity and reliability data for a new PAQ (SP2PAQ) [55]. Twenty-six of the 65 studies were undertaken in the US with the remaining coming from Australia (n = 5), Sweden (n = 5), China (n = 4), Belgium (n = 3), Spain (n = 3), Canada (n = 2), France (n = 2), Norway (n = 2), Japan (n = 2), Brazil, Portugal, Singapore, South Africa, Turkey, United Kingdom and Vietnam. There were four multi-country studies; three testing the IPAQ modified for adolescents [56,57] and the EPAQ-s in 9–10 European cities [51]. The GPAQ was tested in diverse sample of nine global countries [58]. Eighteen studies were undertaken in youth [57,59-74], 12 in elderly [75-86]; and 35 in adults with a few studies including both older adolescents and adults. In 48 studies men and women were combined, 10 studies examined women only [70,72,87-93], and seven studies included only men [54,75,78,94-97]. All authors concluded that the questionnaires had shown at least satisfactory results for reliability and validity (see results below); seven studies noted considerable limitations in aspects of their questionnaires [56,59,63,90,98-100]. 

**Table 5:** Descriptive characteristics of existing PAQs

**Age Group**	**Reference**	**Name questionnaire**	**Country**	**Domains of activity**	**Population**				**Primary outcome**
					***Size***	***Age (years)***	***Sex***	***Ethnicity***	
Youth	Affuso (2011)[59]	SAPAC (modified)	United States	Sedentary	201	11 - 15	M/F	Mixed	Total min/day
Youth	Allor (2001)[60]	PDPAR	United States	Moderate, hard, very hard activity	46	12 ± 0.6	F	Mixed, urban	METs (kcal/hr)
Youth	Corder (2009)[61]	YPAQ, CPAQ, CHASE, SWAPAQ	United Kingdom	All domains, including school and leisure time	62 reliability, 76 validity	4 - 17	M/F	Mainly white	PAEE, lifestyle scores, MET-min/week
Youth	Eisenmann (2002)[62]	GLTEQ	United States	Mild, moderate and strenuous activity in leisure time	31	10.6 ± 0.2	M/F	Mixed	METs
Youth	Gwynn (2010)[63]	MRPARQ	Australia	All organised and non-organised physical activities	86	10 - 12	M/F	Aboriginal, Torres Strait Islander, non-Indigenous	MET-min/day
Youth	Hagströmer (2008)[56]	IPAQ-A	9 countries	Sedentary, leisure, household, occupation, transportation	248	12 - 14, 15 -17	M/F	European	MET-min/day
Youth	Huang (2009)[64]	CLASS (Chinese version)	China	31 physical activities and 14 sedentary activities over weekday and weekends	216 reliability, 99 validity	9 - 12	M/F	Chinese	Total min/day
Youth	Kowalski (1997)[65]	PAQ-C	Canada	Moderate and vigorous PA during school, including sports/exercise	73	8 - 13	M/F	–	5-point scale of activity
Youth	Martinez-Gomez (2010)[66]	BAD	Spain	Leisure, occupation	37	12 - 16	M/F	–	MET-min/day
Youth	Martinez-Gomez (2011)[67]	PAQ-A	Spain	Usual moderate and vigorous PA during schooldays and weekend days	203	13 - 17	M/F	–	PAQ-A score
Youth	Mota (2002)[68]	WAC (modified)	Portugal	Activities outside school	30 reliability, 109 validity	8 - 16	M/F	Hispanic	METs/15 min
Youth	Ottevaere (2011)[57]	IPAQ-A	10 countries	Sedentary, leisure, household, occupation, transportation	2018	12.5 - 17	M/F	European	Total min/day
Youth	Rangul (2008)[69]	HBSC, IPAQ-s	Norway	HBSC: sports/exercise (outside school hours). IPAQ-s: sedentary, leisure, household, occupation, transportation	71	13 - 18	M/F	–	TEE, PAL
Youth	Scerpella (2002)[70]	GSQ	United States	Habitual activity in strenuous, moderate and light intensity	61	7 - 11	F	–	Godin-Shephard scores
Youth	Slinde (2003)[71]	MLTPAQ	Sweden	Sedentary, leisure, household	35	15	M/F	–	TEE
Youth	Treuth (2004)[72]	GAQ	United States	28 physical, 7 sedentary usual activities	90 reliability, 76 comparison validity, 86 intervention validity	8 - 10	F	African-American	GAQ score
Youth	Troped (2007)[73]	YRBS	United States	Leisure, occupation	128 reliability, 125 validity	12.7 ± 0.6	M/F	Mixed	Minutes and bouts of MPA and VPA
Youth	Weston (1997)[74]	PDPAR	United States	Sedentary, leisure, occupation, transportation, sports/exercise	90 reliability, 48 validity	Grades 7 - 12	M/F	Mainly white	METs
Adults	Ainsworth (1999)[87]	TOQ, 7DR-O (modified)	United States	Occupation	46	18 - 60	F	Mainly white	MET-min/week
Adults	Bassett (2000)[101]	CAQ	United States	Stair climbing, walking, sports/exercise, leisure	96	25 - 70	M/F	Mainly Caucasian	MET-min/week
Adults	Brown (2008)[88]	AAS (modified)	Australia	Walking briskly, moderate leisure activity, vigorous leisure activity	44	54 - 59	F	Mainly white	MET-min/week
Adults	Bull (2009)[58]	GPAQ	9 countries	Sedentary, leisure, occupation, transportation	2221 reliability, 298 validity	18-75	M/F	Mixed	Total min/day
Adults	Conway (2002)[94]	7DPAR, S7DR	United States	Household, occupation, walking, light, moderate, vigorous activities	24	27 - 65	M	–	MET-min/day, EE
Adults	Cust (2008)[102]	EPAQ	Australia	Leisure, household, occupation	182	50 - 65	M/F	Mainly white	Total PA index, Cambridge PA index
Adults	Cust (2009)[103]	EPAQ, IPAQ-s	Australia	Sedentary, leisure, household, occupation, transportation	177	50 - 65	M/F	Mainly white	MET-hr/week
Adults	Duncan (2001)[104]	7DPAR	United States	Sedentary, leisure, household, occupation, sports/exercise	94 reliability, 66 validity	30 - 69	M/F	Mainly Caucasian	TEE, METs
Adults	Ekelund (2006)[95]	IPAQ-s	Sweden	Sedentary, leisure, household, occupation, transportation	87	20 - 69	M	–	MET-min/day
Adults	Gauthier (2009)[105]	IPAQ-SALVCF	Canada	Sedentary, leisure, household, occupation, transportation	31	20 - 63	M/F	French Canadians	MET-min/week
Adults	Hagströmer (2006)[106]	IPAQ	Sweden	Sedentary, leisure, household, occupation, transportation	46	40.7 ± 10.3	M/F	–	MET-hr/week
Adults	Hagströmer (2010)[107]	IPAQ	Sweden	Sedentary, leisure, household, occupation, transportation	980	18 - 65	M/F	–	MET-min/day
Adults	Hallal (2010)[108]	IPAQ (modified)	Brazil	Leisure, transportation	156	≥ 20	M/F	–	Total min/week, total score
Adults	InterAct Consortium (2011)[51]	EPAQ-s	10 countries	Leisure, household, occupation, transportation	1941	53.8 ± 9.4	M/F	European	MET-hr/week, total PA index, Cambridge index, recreational index
Adults	Jacobi (2009)[109]	MAQ	France	Sedentary, leisure, occupation	160	18 - 74	M/F	–	MET-hr/week
Adults	Kurtze (2008)[54]	IPAQ-s	Norway	Sedentary, leisure, household, occupation, transportation	108	20 - 39	M	–	MET-hr/week
Adults	Lee (2011)[98]	IPAQ-s (Chinese version)	China	Sedentary, leisure, household, occupation, transportation	1270	42.9 ± 14.4	M/F	Asian	MET-min/week
Adults	MacFarlane (2007)[99]	IPAQ-s (Chinese version)	China	Sedentary, leisure, household, occupation, transportation	49	15 - 55	M/F	Asian	MET-min/week
Adults	MacFarlane (2010)[110]	IPAQ-LC	China	Sedentary, leisure, household, occupation, transportation	28 reliability, 83 validity	26.2 ± 9.9 (reliability), 40.9 ± 11.1 (validity)	M/F	Asian	MET-min/day
Adults	Mahabir (2006)[89]	HAQ, FCPQ, CAPS-4WR, CAPS-TWR	United States	Leisure, household	65	49 - 78	F	–	EE, METs
Adults	Matton (2007)[111]	FPACQ	Belgium	Sedentary, leisure, household, occupation, transportation	102 reliability, 111 validity	22 - 78	M/F	–	Hr/week, EE, PAL (METs)
Adults	Nang (2011)[55]	IPAQ, SP2PAQ	Singapore	Sedentary, leisure, household, occupation, transportation	152	> 21	M/F	Asian	EE (kcal/day), METs
Adults	Nicaise (2011)[90]	IPAQ	United States	Sedentary, leisure, household, occupation, transportation	105	35.9 ± 9.0	F	Latino	MET-min/week
Adults	Pettee-Gabriel (2009)[91]	PMMAQ, PWMAQ, NHS-PAQ, AAS, WHI-PAQ	United States	Sedentary, leisure, sports/exercise	66	45 - 65	F	Mainly white	MET-hr/week, total min/day
Adults	Philippaerts (1999)[96]	BAQ, FCPQ, TCQ	Belgium	Leisure, occupation, sports/exercise	19	40	M	–	PAL scores
Adults	Philippaerts (2001)[97]	BAQ, TCQ	Belgium	Leisure, occupation, sports/exercise	66	40	M	–	Activity indices, EE
Adults	Richardson (2001)[100]	S7DR	United States	Leisure, occupation	77	20 - 59	M/F	Mainly white	MET-min/day
Adults	Saglam (2010)[112]	IPAQ (short and long version)	Turkey	Sedentary, leisure, household, occupation, transportation	330 reliability, 80 validity	18 - 32	M/F	–	MET-min/week
Adults	Schmidt (2006)[92]	KPAS-mod	United States	Household, occupation, active living, sports/exercise	63	18 - 47	F	–	KPAS activity indexes
Adults	Smitherman (2009)[113]	JPAC	United States	Leisure, household, occupation, sports/exercise	40 reliability, 404 validity	54.4 ± 15.7 (reliability), 57.1 ± 11.54 (validity)	M/F	African American	JPAC index scores
Adults	Staten (2001)[93]	AAFQ	United States	Leisure, household, occupation	35	31 - 60	F	Mixed	TEE, PAEE, RMR, MET-hr/day
Adults	Strath (2004)[114]	CAQ-PAI	United States	Leisure	25	20 - 56	M/F	Mainly Caucasian	MET-min/week
Adults	Trinh (2009)[115]	GPAQ	Vietnam	Sedentary, leisure, occupation, transportation	169 dry season, 162 wet season	25 - 64	M/F	Asian	Total min/day
Adults	Washburn (2003)[116]	S7DR	United States	Sleep, moderate, hard and very hard physical activities	46	17 - 35	M/F	Mixed	TEE, PAEE
Adults	Wolin (2008)[117]	IPAQ-s	United States	Sedentary, leisure, household, occupation, transportation	142	24 - 67	M/F	Black or African American	MET-min/week
Elderly	Bonnefoy (2001)[75]	MLTPAQ, YPAS, BAQ-mod, CAQ, 7DR, DQ-mod, LRC, SUA, PASE, QAPSE	France	Light, moderate, vigorous intensity PA, walking, specific activities	19	73.46 ± 4.1	M	–	TEE, PAL, PAEE
Elderly	De Abajo (2001)[76]	YPAS (Spanish version)	Spain	Sedentary, occupation, sports/exercise	108	61 - 80	M/F	Hispanic	Total time, EE
Elderly	Dinger (2004)[77]	PASE	United States	Leisure, household, occupation	56	75.7 ± 7.9	M/F	Mainly Caucasian	Subscale and total PASE scores
Elderly	Dubbert (2004)[78]	7DPAR	United States	Shopping, household, occupation, sports/exercise	220 reliability, 42 validity	60 - 80	M	Mixed	TEE, METs
Elderly	Giles (2009)[79]	CHAMPS-MMSCV	Australia	Leisure, household	47	≥ 65	M/F	Mainly non-Indigenous Australian	MET-min/week (volume), times/week (frequency), min/week (duration)
Elderly	Hagiwara (2008)[80]	PASE	Japan	Leisure, household, occupation	257 reliability, 200 validity	72.6 ± 4.9	M/F	Japanese	Total PASE score, hr/day
Elderly	Harada (2001)[81]	CHAMPS, PASE, YPAS	United States	Leisure, household	87	65 - 89	M/F	Mixed	EE, total PASE score
Elderly	Hurtig-Wennlöf (2010)[82]	IPAQ-E	Sweden	Sedentary, leisure, household, occupation, transportation	54	66 - 85	M/F	–	Total min/day
Elderly	Kolbe-Alexander (2006)[83]	IPAQ-s, YPAS	South Africa	Sedentary, leisure, household, occupation, transportation	122	> 60	M/F	Mixed	MET-min/week, EE
Elderly	Starling (1999)[84]	MLTPAQ, YPAS	United States	MLTPAQ: Leisure, household. YPAS: leisure, household, sports/exercise	67	45 - 84	M/F	Caucasian	TEE
Elderly	Tomioka (2011)[85]	IPAQ-s (Japanese version)	Japan	Sedentary, leisure, household, occupation, transportation	325	65 - 89	M/F	Japanese	MET-min/week
Elderly	Washburn (1999)[86]	PASE	United States	Leisure, household, occupation	20	67 - 80	M/F	–	Total PASE scores

Domains named in paper were reclassified, unless the activities were very different from categories used, according to the following system: Occupation: work, school, labour*.* Transportation: travel, commuting, employment*.* Household: home/life, housework, caregiving, domestic life, child/elder/self care, cooking, chores, gardening, stair climbing. Leisure: leisure, recreation time. Sports/exercise: play, sports, exercise, workout. Sedentary: sedentary behaviours, e.g. sitting, TV viewing activities, eating, sleeping, bathing, inactivity.

– = not stated, M = Male, F = Female.

### Reliability

All reliability results for existing PAQs are listed in Table 6.

**Table 6:** Reliability results of existing PAQs

**Age Group**	**Reference**	**Test-retest period**	**PAQ**	**Variables tested**	**Reliability results**	
					***Correlation coefficients***	***Agreement***
Youth	Allor (2001)[60]	Within 1 week	PDPAR	METs(Q1) – METs(Q2)	ICC = 0.98	–
Youth	Corder (2009)[61]	1 week	YPAQ	12-13 yrs: PAEE(Q1) – PAEE(Q2)	ICC = 0.86 (P < 0.001)	–
				16-17 yrs: PAEE(Q1) – PAEE(Q2)	ICC = 0.79 (P < 0.001)	–
			CPAQ	PAEE(Q1) – PAEE(Q2)	ICC = 0.25	–
			CHASE	Lifestyle score(Q1) – lifestyle score(Q2)	ICC = 0.02	–
			SWAPAQ	PAEE(Q1) – PAEE(Q2)	ICC = 0.64 (P < 0.001)	–
Youth	Eisenmann (2002)[62]	Same day	GLTEQ	Total leisure activity score(Q1) – total leisure activity score(Q2)	Pearson r = 0.62 (P < 0.05)	MD (95 % LoA) = −33.4 ± 10.28
Youth	Huang (2009)[64]	1 week	CLASS	VPA min/week(Q1) – VPA min/week(Q2)	ICC (95 % CI) = 0.73 (0.64;0.79), P < 0.05	–
				MVPA min/week(Q1) – MVPA min/week(Q2)	ICC (95 % CI) = 0.71 (0.61;0.77), P < 0.05	–
				MPA min/week(Q1) – MPA min/week(Q2)	ICC (95 % CI) = 0.61 (0.49;0.70), P < 0.05	–
				Sedentary min/week(Q1) – sedentary min/week(Q2)	ICC (95 % CI) = 0.69 (0.59;0.77), P < 0.05	–
Youth	Mota (2002)[68]	7 days	WAC	Total activity(Q1) – total activity(Q2)	ICC = 0.71	–
Youth	Rangul (2008)[69]	8 - 12 days	HBSC	Frequency: sessions/week(Q1) – sessions/week(Q2)	ICC (95 % CI) = 0.73 (0.60;0.82)	–
				Duration: hr/week(Q1) – hr/week(Q2)	ICC (95 % CI) = 0.71 (0.57;0.81)	–
			IPAQ-s	VPA min/day(Q1) – VPA min/day(Q2)	ICC (95 % CI) = 0.30 (−0.07;0.56)	–
				MPA min/day(Q1) – MPA min/day(Q2)	ICC (95 % CI) = 0.34 (0.22;0.60)	–
				Walking min/day(Q1) – walking min/day(Q2)	ICC (95 % CI) = 0.10 (−0.10;0.39)	–
				Sitting min/day(Q1) – sitting min/day(Q2)	ICC (95 % CI) = 0.27 (−0.50;0.54)	–
Youth	Treuth (2004)[72]	12 weeks	GAQ	Yesterday: GAQ score(Q1) – GAQ score(Q2)	Pearson r = 0.59 (P < 0.001)	–
				Usual: GAQ score(Q1) – GAQ score(Q2)	Pearson r = 0.59 (P < 0.001)	–
				Yesterday: TV watching(Q1) – TV watching(Q2)	Pearson r = 0.13 (P < 0.373)	–
				Usual: TV watching(Q1) – TV watching(Q2)	Pearson r = 0.31 (P < 0.024)	–
				Yesterday: other sedentary(Q1) – other sedentary(Q2)	Pearson r = 0.32 (P < 0.019)	–
				Usual: other sedentary(Q1) – other sedentary(Q2)	Pearson r = 0.30 (P < 0.032)	–
Youth	Troped (2007)[73]	5 - 40 days	YRBS	VPA(Q1) – VPA(Q2)	ICC = 0.46	–
				MPA(Q1) – MPA(Q2)	ICC = 0.51	–
Youth	Weston (1997)[74]	Within 1 hour	PDPAR	TEE(Q1) – TEE(Q2)	Pearson r = 0.98 (P < 0.01)	–
Adults	Brown (2008)[88]	7 - 28 days	AAS	Frequency/week(Q1) – frequency/week(Q2)	Spearman r = 0.58	–
				Total min/week(Q1) – total min/week(Q2)	Spearman r = 0.64	–
Adults	Bull (2009)[58]	3 - 7 days	GPAQ	Leisure: total min(Q1) – total min(Q2)	Spearman r = 0.78 (P < 0.01)	–
				Occupation: total min(Q1) – total min(Q2)	Spearman r = 0.77 (P < 0.01)	–
				Transportation: total min(Q1) – total min(Q2)	Spearman r = 0.81 (P < 0.01)	–
				Leisure: sedentary(Q1) – sedentary(Q2)	–	κ (% agreement) = 0.68 (85.6)
				Occupation: sedentary(Q1) – sedentary(Q2)	–	κ (% agreement) = 0.73 (86.9)
Adults	Cust (2008)[102]	10 months	EPAQ	Total MET-hr/week(Q1) – total MET-hr/week(Q2)	Spearman r (95 % CI) = 0.65 (0.55;0.72), P < 0.0001	–
				Total PA index(Q1) – total PA index(Q2)	–	κ (95 % CI) = 0.62 (0.53;0.71), P < 0.0001
				Cambridge PA index(Q1) – Cambridge PA index(Q2)	–	κ (95 % CI) = 0.66 (0.58;0.74), P < 0.0001
Adults	Cust (2009)[103]	10 months	EPAQ	High confidence: total PA index(Q1) – total PA index(Q2)	–	κ (95 % CI) = 0.65 (0.53;0.76)
				Low confidence: total PA index(Q1) – total PA index(Q2)	–	κ (95 % CI) = 0.58 (0.45;0.71)
				High confidence: Cambridge PA index(Q1) – Cambridge PA index(Q2)	–	κ (95 % CI) = 0.73 (0.61;0.84)
				Low confidence: Cambridge PA index(Q1) – Cambridge PA index(Q2)	–	κ (95 % CI) = 0.59 (0.47;0.71)
			IPAQ-s	High confidence: total MET-hr/week(Q1) – total MET-hr/week(Q2)	Spearman r (95 % CI) = 0.53 (0.36;0.67)	–
				Low confidence: total MET-hr/week(Q1) – total MET-hr/week(Q2)	Spearman r (95 % CI) = 0.33 (0.11;0.52)	–
				High confidence: sitting hr/day(Q1) – sitting hr/day(Q2)	Spearman r (95 % CI) = 0.50 (0.32;0.65)	–
				Low confidence: sitting hr/day(Q1) – sitting hr/day(Q2)	Spearman r (95 % CI) = 0.65 (0.51;0.75)	–
Adults	Duncan (2001)[104]	7 days	7DPAR	TEE(Q1) – TEE(Q2)	ICC (95 % CI) = 0.44 (0.26;0.59)	–
Adults	Gauthier (2009)[105]	1 day	IPAQ-SALVCF	Total MET-min/week(Q1) – total MET-min/week(Q2)	ICC (95 % CI) = 0.929 (0.860;0.965), P < 0.01	–
				Sitting(Q1) – sitting(Q2)	ICC (95 % CI) = 0.899 (0.800;0.950), P < 0.01	–
Adults	Hallal (2010)[108]	5 days	IPAQ	Total score(T1) – total score(T2)	Spearman r = 0.90	MD = 3 min, κ (% agreement) = 0.78 (90.0)
				Total score(T1T2) – total score(FTF)	Spearman r = 0.87	MD = 30 min, κ (% agreement) = 0.69 (85.5)
Adults	Kurtze (2008)[54]	1 week	IPAQ-s	VPA hr/day(Q1) – VPA hr/day(Q2)	ICC (95 % CI) = 0.62 (0.47;0.73)	–
				MPA hr/day(Q1) – MPA hr/day(Q2)	ICC (95 % CI) = 0.30 (0.09;0.49)	–
				Walking hr/day(Q1) – walking hr/day(Q2)	ICC (95 % CI) = 0.42 (0.23;0.59)	–
				Sitting hr/day(Q1) – sitting hr/day(Q2)	ICC (95 % CI) = 0.80 (0.70;0.87)	–
Adults	MacFarlane (2007)[99]	3 days	IPAQ-s	Total MET-min/week(Q1) – total MET-min/week(Q2)	ICC (95 % CI) = 0.79 (0.66;0.88), %CV (95 % CI) = 26 (22;33)	–
				Sitting MET-min/week(Q1) – sitting MET-min/week(Q2)	ICC (95 % CI) = 0.97 (0.95;0.98), %CV (95 % CI) = 15 (12;18)	–
Adults	MacFarlane (2010)[110]	3 days	IPAQ-LC	Total MET-min/week(Q1) – total MET-min/week(Q2)	ICC = 0.93, %CV = 22.8	–
				Sitting MET-min/week(Q1) – sitting MET-min/week(Q2)	ICC = 0.71, %CV = 15.0	–
Adults	Matton (2007)[111]	2 weeks	FPACQ	Employed/unemployed men: total EE(Q1) – total EE(Q2)	ICC (95 % CI) = 0.95 (0.89;0.97)	–
				Employed/unemployed women: total EE(Q1) – total EE(Q2)	ICC (95 % CI) = 0.92 (0.85;0.96)	–
				Retired men: total EE(Q1) – total EE(Q2)	ICC (95 % CI) = 0.90 (0.76;0.96)	–
				Retired women: total EE(Q1) – total EE(Q2)	ICC (95 % CI) = 0.96 (0.90;0.99)	–
				Employed/unemployed men: PAL(Q1) – PAL(Q2)	ICC (95 % CI) = 0.92 (0.84;0.96)	–
				Employed/unemployed women: PAL(Q1) – PAL(Q2)	ICC (95 % CI) = 0.78 (0.61;0.88)	–
				Retired men: PAL(Q1) – PAL(Q2)	ICC (95 % CI) = 0.89 (0.76;0.96)	–
				Retired women: PAL(Q1) – PAL(Q2)	ICC (95 % CI) = 0.77 (0.47;0.91)	–
				Employed/unemployed men: TV hr/week(Q1) – TV hr/week(Q2)	ICC (95 % CI) = 0.93 (0.86;0.97)	–
				Employed/unemployed women: TV hr/week(Q1) – TV hr/week(Q2)	ICC (95 % CI) = 0.92 (0.84;0.96)	–
				Retired men: TV hr/week(Q1) – TV hr/week(Q2)	ICC (95 % CI) = 0.76 (0.49;0.89)	–
				Retired women: TV hr/week(Q1) – TV hr/week(Q2)	ICC (95 % CI) = 0.89 (0.72;0.96)	–
Adults	Nang (2011)[55]	2 - 10 months	IPAQ	VPA(Q1) – VPA(Q2)	Spearman r = 0.38 (P < 0.05)	–
				MPA(Q1) – MPA(Q2)	Spearman r = 0.58 (P < 0.0001)	–
			SP2PAQ	VPA(Q1) – VPA(Q2)	Spearman r = 0.75 (P < 0.0001)	–
				MPA(Q1) – MPA(Q2)	Spearman r = 0.55 (P < 0.0001)	–
Adults	Pettee-Gabriel (2009)[91]	1 - 4 weeks	PMMAQ	MET-hr/week(Q1) – MET-hr/week(Q2)	ICC (95 % CI) = 0.64 (0.48;0.77), P < 0.0001	–
			PWMAQ	MET-hr/week(Q1) – MET-hr/week(Q2)	ICC (95 % CI) = 0.74 (0.60;0.83), P < 0.0001	–
			NHS-PAQ	MET-hr/week(Q1) – MET-hr/week(Q2)	ICC (95 % CI) = 0.48 (0.26;0.65), P < 0.0001	–
			AAS	Min/day(Q1) – min/day(Q2)	ICC (95 % CI) = 0.32 (0.09;0.52), P < 0.01	–
			WHI-PAQ	MET-hr/week(Q1) – MET-hr/week(Q2)	ICC (95 % CI) = 0.91 (0.86;0.95), P < 0.0001	–
Adults	Richardson (2001)[100]	1 month	S7DR	Men: total MET-min/day(Q1) – total MET-min/day(Q2)	Spearman r = 0.60 (P < 0.01)	–
				Women: total MET-min/day(Q1) – total MET-min/day(Q2)	Spearman r = 0.36 (P < 0.05)	–
Adults	Saglam (2010)[112]	3 - 7 days	IPAQ	Total MET-min/week(Q1) – total MET-min/week(Q2)	Spearman r (95 % CI) = 0.64 (0.56;0.72), P < 0.001	–
				Sitting min(Q1) – sitting min(Q2)	Spearman r (95 % CI) = 0.83 (0.77;0.89), P < 0.001	–
			IPAQ-s	Total MET-min/week(Q1) – total MET-min/week(Q2)	Spearman r (95 % CI) = 0.69 (0.61;0.77), P < 0.001	–
				Sitting min(Q1) – sitting min(Q2)	Spearman r (95 % CI) = 0.78 (0.71;0.85), P < 0.001	–
Adults	Schmidt (2006)[92]	7 days	KPAS-mod	Total activity score(Q1) – total activity score(Q2)	ICC = 0.84	–
				Weighted activity score(Q1) – weighted activity score(Q2)	ICC = 0.76	–
Adults	Smitherman (2009)[113]	2 weeks	JPAC	JPAC total score(Q1) – JPAC total score(Q2)	ICC = 0.99	–
Adults	Trinh (2009)[115]	2 weeks (dry season)	GPAQ	GPAQ total score(Q1) – GPAQ total score(Q2)	Spearman r = 0.69 (P < 0.001)	MD (95 % LoA) = 1.00 (0.03;31.82), κ (95 % CI) = 0.66 (0.53;0.79)
		2 months (wet season)		GPAQ total score(Q1) – GPAQ total score(Q2)	Spearman r = 0.55 (P < 0.001)	MD (95 % LoA) = 1.12 (0.02;71.09), κ (95 % CI) = 0.57 (0.46;0.65)
		2 weeks (dry season)		Sedentary time(Q1) – sedentary time(Q2)	Spearman r = 0.69 (P < 0.001)	κ (95 % CI) = 0.61 (0.58;0.70)
		2 months (wet season)		Sedentary time(Q1) – sedentary time(Q2)	Spearman r = 0.50 (P < 0.001)	κ (95 % CI) = 0.45 (0.36;0.54)
Elderly	De Abajo (2001)[76]	2 weeks	YPAS	Total time(Q1) – total time(Q2)	ICC = 0.66 (P = 0.001)	–
				Total EE(Q1) – total EE(Q2)	ICC = 0.65 (P = 0.001)	–
				YPAS summary index(Q1) – YPAS summary index(Q2)	ICC = 0.31 (P = 0.002)	–
				Sitting(Q1) – sitting(Q2)	ICC = 0.29 (P = 0.003)	–
Elderly	Dinger (2004)[77]	3 days	PASE	Total PASE score(Q1) – total PASE score(Q2)	ICC (95 % CI) = 0.91 (0.83;0.94)	–
Elderly	Dubbert (2004)[78]	2 - 4 weeks	7DPAR	TEE(Q1) – TEE(Q2)	ICC = 0.89 (P < 0.001)	–
Elderly	Giles (2009)[79]	1 - 2 weeks	CHAMPS-MMSCV	Volume: MET-min/week(Q1) – MET-min/week(Q2)	ICC (95 % CI) = 0.84 (0.69;0.91), Spearman r = 0.62	–
				Frequency: sessions/week(Q1) – sessions/week(Q2)	ICC (95 % CI) = 0.89 (0.77;0.95), Spearman r = 0.79	–
				Duration: min/week(Q1) – min/week(Q2)	ICC (95 % CI) = 0.81 (0.63;0.90), Spearman r = 0.57	–
Elderly	Hagiwara (2008)[80]	3 - 4 weeks	PASE	Total PASE score(Q1) – total PASE score(Q2)	ICC (95 % CI) = 0.65 (0.58;0.72)	–
Elderly	Harada (2001)[82]	2 weeks	CHAMPS	EE(Q1) – EE(Q2)	ICC = 0.62, Pearson r = 0.62	–
Elderly	Kolbe-Alexander (2006)[83]	3 - 5 days	IPAQ-s	Men: total MET-min/week(Q1) – total MET-min/week(Q2)	Spearman r = 0.54 (P = 0.0001)	MD (95 % LoA) = 324.58 ± 7534.85 MET-min/week
				Women: total MET-min/week(Q1) – total MET-min/week(Q2)	Spearman r = 0.60 (P = 0.0000)	MD (95 % LoA) = 347.14 ± 4016.88 MET-min/week
				Men: sitting MET-hr/week(Q1) – sitting MET-hr/week(Q2)	Spearman r = 0.76 (P = 0.0000)	–
				Women: sitting MET-hr/week(Q1) – sitting MET-hr/week(Q2)	Spearman r = 0.77 (P = 0.0000)	–
			YPAS	Men: total MET-min/week(Q1) – total MET-min/week(Q2)	Spearman r = 0.57 (P = 0.00001)	MD (95 % LoA) = −582.17 ± 4867.14 MET-min/week
				Women: total MET-min/week(Q1) – total MET-min/week(Q2)	Spearman r = 0.62 (P = 0.0000)	MD (95 % LoA) = 26.77 ± 4474.64 MET-min/week
Elderly	Tomioka (2011)[85]	2 weeks	IPAQ-s	Young old men: MET-min/week(Q1) – MET-min/week(Q2)	ICC (95 % CI) = 0.65 (0.46;0.78)	–
				Young old women: MET-min/week(Q1) – MET-min/week(Q2)	ICC (95 % CI) = 0.57 (0.34;0.72)	–
				Old old men: MET-min/week(Q1) – MET-min/week(Q2)	ICC (95 % CI) = 0.50 (0.22;0.68)	–
				Old old women: MET-min/week(Q1) – MET-min/week(Q2)	ICC (95 % CI) = 0.56 (0.30;0.72)	–
				Young old men: sitting hr/day(Q1) – sitting hr/day(Q2)	ICC (95 % CI) = 0.82 (0.71;0.88)	–
				Young old women: sitting hr/day(Q1) – sitting hr/day(Q2)	ICC (95 % CI) = 0.70 (0.54;0.80)	–
				Old old men: sitting hr/day(Q1) – sitting hr/day(Q2)	ICC (95 % CI) = 0.66 (0.48;0.78)	–
				Old old women: sitting hr/day(Q1) – sitting hr/day(Q2)	ICC (95 % CI) = 0.67 (0.48;0.80)	–
					*Median ICC = 0.71 (youth: 0.64, adults: 0.79, elderly: 0.65)*	
					*Median Spearman r = 0.62 (youth: –, adults: 0.64, elderly: 0.60)*	
					*Median Pearson r = 0.62 (youth: 0.605, adults: –, elderly: 0.62)*	
						*Median κ = 0.655 (youth: –, adults: 0.655, elderly: –)*

Q1 = first completed questionnaire, Q2 = second completed questionnaire, r = correlation coefficient (rho), ICC = Intraclass Correlation Coefficient, CI = Confidence Interval (lower;upper), %CV = coefficient of variation (within subjects standard deviation of typical error) as a percentage of the mean score, κ = kappa (i.e. Cohen weighted kappa unless specified otherwise), LoA = Limits of Agreement, MD = Mean Difference, – = not stated.

Bull (2009): Total min = total time per domain of the pooled data (n = 2221) of 7 countries (Bangladesh, China, Ethiopia, Indonesia, South Africa, Japan, Taiwan). Leisure = discretionary domain, occupation = work domain, transportation = transport domain. Sedentary = categorical variable of pooled data (n = 1524) for no physical activity in the discretionary or work domain.

Corder (2009): PAEE in kJ/kg/day for total group, or for 12 – 13 or 16 – 17 year old children. Lifestyle score = summed score of four multiple choice questions regarding active transport, school break activities, activity outside school, and the amount of "exercise that makes you out of breath".

Cust (2008): Total MET-hr/week = total MET hours per week of non-occupational activity. Total PA index = cross-tabulation of level of occupational activity with combined recreational and household activities - inactive, moderately inactive, moderately active, active. Cambridge PA index = index based on occupational, cycling and sports activity (generally more intense activities).

Cust (2009): Results are stratified according to the group of participants reporting high or low confidence in recall of PA. High confidence = group of participants reporting high self-reported confidence in recall of physical activity. Low confidence = group of participants reporting low self-reported confidence in recall of physical activity.

De Abajo (2001): EE in kJ/day. YPAS summary index = summed time for each activity, expressed in hours per week for each subject. Individual indices were created by multiplying a frequency score by a duration score and multiplying again by a weighting factor.

Dinger (2004): Total PASE score = weighted and summed score of individual items using the PASE scoring algorithm.

Eisenmann (2002): Same day = beginning and end of the day. Total leisure activity score was calculated by multiplying the frequency of each category by the MET value and summing the score.

Gauthier (2009): Total MET-min/week = total activity excluding sitting.

Hagiwara (2008): PASE score was calculated by adding the score for each component determined on the basis of the time spent on each activity or the presence or absence of activity over the past 7 days. In the paper more details (κ or weighted κ and the proportion of consistency) are reported for each separate activity component.

Hallal (2010): Total score = sum of minutes spent on MPA (including walking) per week, and twice the number of minutes spent on VPA. T1 = telephone interview on day 1. T2 = telephone interview on day 6. FTF = face-to-face interview on day 1.

Harada (2001): EE in kcal/week.

Huang (2009): Activity intensities classified according to a compendium of physical activities.

Kolbe-Alexander (2006): sitting = time spent sitting during a week and weekend day.

Kurtze (2008): VPA = 8 METs, MPA = 4 METs, Walking = 3.3 METs on average.

MacFarlane (2007/2010): Total MET-min/week = total activity excluding sitting (1 MET).

Matton (2007): EE in kcal/week. PAL is calculated as total EE divided by 168 (number of hours per week) and the reported body weight. TV hr/week = time per week spent watching television or videos or playing computer games during weekdays and weekends.

Nang (2011): VPA(Q) = 3–6 METs kcal/day, MPA(Q) = >6 METs kcal/day.

Pettee-Gabriel (2009): Test-retest period = 1 week for PWMAQ (n = 65), NHS-PAQ (n = 62), AAS (n = 65), WHI-PAQ (n = 63) and 1 month for PMMAQ (n = 65).

Schmidt (2006): Total activity score = activity score of all four domains, calculated as: (household/caregiving index*0.25 + occupational index*0.25 + active living index*0.25 + sports/exercise index*0.25)*4. Weighted activity score = activity score of all four domains, calculated as: (household/caregiving index*0.50 + occupational index*0.20 + active living index*0.25 + sports/exercise index*0.05)*4.

Smitherman (2009): JPAC total score = total score calculated by summing the 4 index scores (active living, work, home/family/yard/garden, sport/exercise index) and can range from 3 to 20.

Tomioka (2011): Young old = age 65–74, old old = age 75–89.

Treuth (2004): GAQ score yesterday = summary score estimated from 28 physical activities performed on the previous day (yesterday), applying the code 0 for the response "none", 1 for the response "less than 15 min", and 10 for the response "15 min or more". GAQ score usual = summary score estimated from usual activities, based on frequency of physical activities performed, applying the code 0 for the response "none", 1 for the response "a little", and 10 for the response "a lot". The GAQ summary scores were computed as the total MET-weighted score divided by the number of nonmissing items. TV watching = time spent watching TV or video. Other sedentary = time spent performing computer or video games, arts and crafts, board games, homework or reading, talking on phone or hanging out.

Trinh (2009): GPAQ total score = score of 19 items following the GPAQ analysis protocol. Sedentary time = time spent sitting or reclining. MD (95 % LoA) = log-transformed average difference with 95 % limits of agreement. Compared with the baseline assessment, the GPAQ score was on average not different and 12 % higher, respectively, 2 weeks later.

Troped (2007): MPA = number of days participating in ≥ 30 min of moderate PA during past 7 days. VPA = number of days participating in ≥ 20 min of vigorous PA during past 7 days.

Weston (1997): TEE in kcal/kg/day.

Most studies examining the reliability of existing PAQs reported reliability as ICC (n = 20), Pearson/Spearman correlation coefficients (n = 8); some studies also used a combination of correlation statistics (n = 7). Similar to the new PAQs, the existing PAQs demonstrated moderate correlations for reliability. Median correlations from reported data for recall of sedentary behaviours were divergent: ICC = 0.76, Spearman r = 0.725, Pearson r = 0.305, kappa = 0.645.

### Youth

Median reliability correlations for the youth were as follows: ICC = 0.64, Pearson r = 0.605. The CHASE (ICC = 0.02) and the CPAQ (ICC = 0.25) showed poor test-retest reliability, whereas the reliability was strong for YPAQ (ICC = 0.79–0.86) in the same study [61]. Previous day physical activity recall instruments proved to be highly reliable in children (ICC = 0.98 [60], r = 0.98 [74]).

### Adults

Median reliability correlations for adults were as follows: ICC = 0.79, Spearman r = 0.64, kappa = 0.655. The IPAQ-SALVCF (ICC = 0.929) [105], IPAQ long version (r = 0.87–0.90 [108], ICC = 0.93 [110]), IPAQ short version (ICC = 0.79) [99], FPACQ (ICC = 0.77–0.96) [111], KPAS-mod (ICC = 0.76–0.84) [92] and the JPAC (ICC = 0.99) [113] showed acceptable or strong reliability. Notably, the IPAQ-s showed a wide range of results for reliability, with ICCs ranging from 0.27–0.97 for sitting [54,69,83,85,99,103,112], 0.10–0.42 for walking [54,69], 0.30–0.34 for MPA [54,69], 0.30–0.62 for VPA [54,69], and 0.33–0.79 for total PA [83,85,99,103,112]. For sedentary time the short IPAQ appeared to be the most reliable questionnaire when the test retest duration was short (i.e. 3 days, [ICC = 0.97]) [99]. All existing PAQs for adults reported acceptable to high reliability properties, overall.

### Elderly

Median reliability correlations for the elderly were as follows: ICC = 0.65, Spearman r = 0.60, Pearson r = 0.62. Similarly, all existing PAQs for elderly also showed overall acceptable to high reliability, with the PASE (ICC = 0.91) [77], 7DPAR (ICC = 0.89) [78] and CHAMPS-MMSCV (ICC = 0.81–0.89) [79] performing best.

### Validity

All validity results for existing PAQs are listed in Table 7.

**Table 7:** Validity results of existing PAQs

**Age Group**	**Reference**	**Criterion method**	**Duration of validation**	**PAQ**	**Variables tested**	**Criterion intensity thresholds**	**Validity results**	
							***Correlation coefficients***	***Agreement***
Youth	Affuso (2011)[59]	Acc (ActiGraph)	3 days	SAPAC	Sedentary mins(Q) – sedentary mins(Acc)	<100 counts/min	Pearson r (95 % CI) = 0.18 (0.07;0.28), Spearman r (95 % CI) = 0.14 (0.05;0.23)	–
Youth	Allor (2001)[60]	Acc (Caltrac)	2 days	PDPAR	EE(Q) – EE(Acc)	–	Pearson r = 0.76 (P < 0.01)	MD = ~100 kcal/hr (P < 0.01)
		HR	2 days		EE(Q) – EE(HR)	–	Pearson r = 0.50 (P < 0.01)	MD = ~100 kcal/hr
Youth	Corder (2009)[61]	DLW	11 days	YPAQ	12-13 yrs: PAEE(Q) – PAEE(DLW)	–	Spearman r = 0.09 (P = 0.67)	MD (95 % LoA) = 0.59 ± 6.3 kJ/kg/day
					16-17 yrs: PAEE(Q) – PAEE(DLW)	–	Spearman r = 0.46 (P = 0.03)	MD (95 % LoA) = 0.32 ± 4.6 kJ/kg/day
		Acc (ActiGraph)	11 days		12-13 yrs: MVPA(Q) – MVPA(Acc)	≥1952 counts/min	Spearman r = 0.42 (P = 0.04)	MD (95 % LoA) = 2.01 ± 2.25 min/week
					16-17 yrs: MVPA(Q) – MVPA(Acc)	≥1952 counts/min	Spearman r = 0.11 (P = 0.61)	MD (95 % LoA) = 1.38 ± 2.97 min/week
		DLW	11 days	CPAQ	PAEE(Q) – PAEE(DLW)	–	Spearman r = 0.22 (P = 0.28)	MD (95 % LoA) = 0.76 ± 3.1 kJ/kg/day
		Acc (ActiGraph)	11 days		MVPA(Q) – MVPA(Acc)	≥1952 counts/min	Spearman r = 0.42 (P = 0.04)	MD (95 % LoA) = 1.63 ± 2.24 min/week
		DLW	11 days	CHASE	Lifestyle score(Q) – PAEE(DLW)	–	Spearman r = 0.45 (P = 0.02)	–
		Acc (ActiGraph)	11 days		Lifestyle score(Q) – MVPA(Acc)	≥1952 counts/min	Spearman r = 0.12 (P = 0.57)	–
		DLW	11 days	SWAPAQ	PAEE(Q) – PAEE(DLW)	–	Spearman r = 0.40 (P = 0.04)	MD (95 % LoA) = 0.46 ± 8.5 kJ/kg/day
		Acc (ActiGraph)	11 days		MVPA(Q) – MVPA(Acc)	≥1952 counts/min	Spearman r = 0.23 (P = 0.27)	MD (95 % LoA) = 1.03 ± 2.58 min/week
Youth	Eisenmann (2002)[62]	Acc (Caltrac)	1 day	GLTEQ	Total leisure activity score(Q) – counts/hr(Acc)	–	Pearson r = 0.50	–
Youth	Gwynn (2010)[63]	Acc (ActiGraph)	7 days	MRPARQ	MVPA min/day(Q) – MVPA min/day(Acc)	≥1952 counts/min	Pearson r = 0.37 (P < 0.05), ICC = 0.25 (P < 0.05)	–
Youth	Hagströmer (2008)[56]	Acc (ActiGraph)	7 days	IPAQ-A	Total MET-min/day(Q) – total counts/min(Acc)	–	Spearman r = 0.20 (P < 0.01)	–
Youth	Huang (2009)[64]	Acc (ActiGraph)	7 days	CLASS	Boys: VPA min/week(Q) – VPA min/week(Acc)	≥6 METs	Spearman r = 0.29	MD (95 % LoA) = 12.6 ± 47.4 min/week
					Girls: VPA min/week(Q) – VPA min/week(Acc)	≥6 METs	Spearman r = 0.43 (P < 0.05)	MD (95 % LoA) = 12.6 ± 47.4 min/week
					Boys: MVPA min/week(Q) – MVPA min/week(Acc)	≥3 METs	Spearman r = 0.27	MD (95 % LoA) = −6.2 ± 95.3 min/week
					Girls: MVPA min/week(Q) – MVPA min/week(Acc)	≥3 METs	Spearman r = 0.48 (P < 0.05)	MD (95 % LoA) = −6.2 ± 95.3 min/week
					Boys: MPA min/week(Q) – MPA min/week(Acc)	3-5.9 METs	Spearman r = 0.33	MD (95 % LoA) = −18.9 ± 70.4 min/week
					Girls: MPA min/week(Q) – MPA min/week(Acc)	3-5.9 METs	Spearman r = 0.29 (P < 0.05)	MD (95 % LoA) = −18.9 ± 70.4 min/week
					Boys: sedentary min/week(Q) – sedentary min/week(Acc)	<100 counts/min	Spearman r = 0.06	–
					Girls: sedentary min/week(Q) – sedentary min/week(Acc)	<100 counts/min	Spearman r = 0.25 (P < 0.05)	–
Youth	Kowalski (1997)[65]	Acc (Caltrac)	7 days	PAQ-C	PAQ-C score(Q) – total counts(Acc)	–	Pearson r = 0.39 (P < 0.05)	–
Youth	Martinez-Gomez (2010)[66]	Acc (ActiGraph)	3 days	BAD	Total MET-min/day(Q) – total counts/day(Acc)	–	Spearman r = 0.29	–
					Total MET-min/day(Q) – total counts/min/day(Acc)	–	Spearman r = 0.33	–
Youth	Martinez-Gomez (2011)[67]	Acc (ActiGraph)	7 days	PAQ-A	PAQ-A score(Q) – total counts/min(Acc)	–	Spearman r = 0.39 (P < 0.001)	–
					PAQ-A score(Q) – MVPA mins(Acc)	≥1952 counts/min	Spearman r = 0.31 (P < 0.001)	–
Youth	Mota (2002)[68]	Acc (ActiGraph)	3 days	WAC	METs/15 min(Q) – counts/min(Acc)	–	Pearson r = 0.30 (P = 0.01)	–
Youth	Ottevaere (2011)[57]	Acc (ActiGraph)	7 days	IPAQ-A	VPA min/day(Q) – VPA min/day(Acc)	≥4000 counts/min	Spearman r = 0.25 (P < 0.01)	MD (95 % LoA) = 13.2 ± 78.2 min/day
					MVPA min/day(Q) – MVPA min/day(Acc)	≥2000 counts/min	Spearman r = 0.21 (P < 0.01)	–
					MPA min/day(Q) – MPA min/day(Acc)	2000-3999 counts/min	Spearman r = 0.15 (P < 0.01)	MD (95 % LoA) = 31.6 ± 105.6 min/day
Youth	Rangul (2008)[69]	Acc (ActiReg)	7 days	HBSC	Frequency(Q) – TEE(Acc)	–	Spearman r = 0.20	–
					Frequency(Q) – PAL(Acc)	–	Spearman r = 0.02	–
					Duration(Q) – TEE(Acc)	–	Spearman r = 0.23	–
					Duration(Q) – PAL(Acc)	–	Spearman r = 0.01	–
				IPAQ-s	VPA min/day(Q) – TEE(Acc)	–	Spearman r = −0.14	–
					VPA min/day(Q) – PAL(Acc)	–	Spearman r = −0.08	–
					MPA min/day(Q) – TEE(Acc)	–	Spearman r = 0.01	–
					MPA min/day(Q) – PAL(Acc)	–	Spearman r = 0.01	–
					Walking min/day(Q) – TEE(Acc)	–	Spearman r = 0.24	–
					Walking min/day(Q) – PAL(Acc)	–	Spearman r = 0.43 (P < 0.01)	–
					Sitting min/day(Q) – TEE(Acc)	–	Spearman r = −0.04	–
					Sitting min/day(Q) – PAL(Acc)	–	Spearman r = −0.29	–
Youth	Scerpella (2002)[70]	Acc (Caltrac)	2x 3 days	GSQ	Godin-Shephard score(Q) – Caltrac score(Acc)	–	Spearman r = 0.102 (P = 0.422)	–
Youth	Slinde (2003)[71]	DLW	14 days	MLTPAQ	TEE(Q) – TEE(DLW)	–	Spearman r = 0.49 (P < 0.01)	–
				eMLTPAQ	TEE(Q) – TEE(DLW)	–	Spearman r = 0.65 (P < 0.01)	MD (95 % LoA) = 2.8 ± 2.8 MJ/day
					Sedentary min/day(Q) – TEE(DLW)	–	Spearman r = 0.030 (P = 0.86)	–
Youth	Treuth (2004)[72]	Acc (ActiGraph)	3 days	GAQ	Baseline: yesterday GAQ score(Q) – mean counts/min(Acc)	–	Pearson r = 0.06 (P = 0.42)	–
					Follow-up: yesterday GAQ score(Q) – mean counts/min(Acc)	–	Pearson r = 0.08 (P = 0.28)	–
					Baseline: usual GAQ score(Q) – mean counts/min(Acc)	–	Pearson r = 0.12 (P = 0.10)	–
					Follow-up: usual GAQ score(Q) – mean counts/min(Acc)	–	Pearson r = 0.07 (P = 0.36)	–
Youth	Troped (2007)[73]	Acc (ActiGraph)	7 days	YRBS	Total VPA min/day(Q) – total VPA min/day(Acc)	>6 METs	Sensitivity = 0.86, specificity: 0.26	κ = −0.002 – 0.06
					Total MPA min/day(Q) – total MPA min/day(Acc)	3-6 METs	Sensitivity = 0.23, specificity: 0.92	κ = −0.05 – 0.03
Youth	Weston (1997)[74]	Acc (Caltrac)	1 day (after school)	PDPAR	TEE(Q) – total counts(Acc)	–	Pearson r = 0.77 (P < 0.01)	–
		HR (Polar)	1 day (after school)		EE(Q) – %HRR(HR)	–	Pearson r = 0.53 (P < 0.01)	–
Adults	Ainsworth (1999)[87]	Acc (Caltrac)	7 days	TOQ	MPA MET-min/week(Q) – EE(Acc)	–	Pearson r = 0.34 (P < 0.05)	–
				7DR-O	7DR scores(Q) – EE(Acc)	–	Low correlations (P > 0.05)	–
Adults	Bassett (2000)[101]	Ped (Yamax)	7 days	CAQ	Men: distance(Q) – distance(Ped)	–	r = 0.346 (P = 0.02)	–
					Women: distance(Q) – distance(Ped)	–	r = 0.481 (P = 0.001)	–
Adults	Brown (2008)[88]	Acc (ActiGraph)	7 days	AAS	Frequency/week(Q) – frequency(Acc)	≥3 METs, ≥1952 counts/min	Spearman r = 0.48 (P = 0.001)	–
					Total min/week(Q) – MVPA(Acc)	≥3 METs, ≥1952 counts/min	Spearman r = 0.52 (P < 0.001)	–
					Total min/week(Q) – total counts(Acc)	–	Spearman r = 0.23 (P = 0.14)	–
Adults	Bull (2009)[58]	Acc (MTI)	> 7 days	GPAQ	China: VPA(Q) – mean VPA counts/day(Acc)	–	Spearman r = 0.23 (P < 0.05)	–
					South Africa: VPA(Q) – mean VPA counts/day(Acc)	–	Spearman r = 0.26 (P < 0.05)	–
					China: MPA(Q) – mean MPA counts/day(Acc)	–	Spearman r = 0.23 (P < 0.05)	–
					South Africa: MPA(Q) – mean MPA counts/day(Acc)	–	Spearman r = −0.03	–
					China: sedentary min/day(Q) – mean sedentary counts/day(Acc)	<100 counts/min	Spearman r = 0.40 (P < 0.05)	–
					South Africa: sedentary min/day(Q) – mean sedentary counts/day(Acc)	<100 counts/min	Spearman r = −0.02	–
Adults	Conway (2002)[94]	DLW	14 days	7DPAR	TEE(Q) – TEE(DLW)	–	R^2^ = 0.10	MD (±SEM) = 0.91 ± 0.42 (7.9 ± 3.2 %) MJ/day
				S7DR	TEE(Q) – TEE(DLW)	–	R^2^ = 0.14	MD (±SEM) = 4.14 ± 1.36 (30.6 ± 9.9 %) MJ/day
Adults	Cust (2008)[102]	Acc (ActiGraph)	3x 7 days	EPAQ	Total MET-hr/week(Q) – total MET-hr/week(Acc)	≥574 counts/min	Spearman r (95 % CI) = 0.21 (0.07;0.35), P < 0.01	–
					Total PA index(Q) – total MET-hr/week(Acc)	≥574 counts/min	Spearman r (95 % CI) = 0.29 (0.15;0.42), P < 0.0001	–
					Cambridge PA index(Q) – total MET-hr/week(Acc)	≥574 counts/min	Spearman r (95 % CI) = 0.32 (0.19;0.45), P < 0.0001	–
Adults	Cust (2009)[103]	Acc (ActiGraph)	3x 7 days	EPAQ	High confidence: total PA index(Q) – total MET-hr/week(Acc)	≥574 counts/min	Spearman r (95 % CI) = 0.37 (0.17;0.54)	–
					Low confidence: total PA index(Q) – total MET-hr/week(Acc)	≥574 counts/min	Spearman r (95 % CI) = 0.22 (0.02;0.41)	–
					High confidence: Cambridge PA index(Q) – total MET-hr/week(Acc)	≥574 counts/min	Spearman r (95 % CI) = 0.30 (0.10;0.48)	–
					Low confidence: Cambridge PA index(Q) – total MET-hr/week(Acc)	≥574 counts/min	Spearman r (95 % CI) = 0.35 (0.15;0.52)	–
				IPAQ-s	High confidence: total MET-hr/week(Q) – total MET-hr/week(Acc)	≥574 counts/min	Spearman r (95 % CI) = 0.26 (0.04;0.45)	–
					Low confidence: total MET-hr/week(Q) – total MET-hr/week(Acc)	≥574 counts/min	Spearman r (95 % CI) = 0.27 (0.07;0.46)	–
					High confidence: sitting hr/day(Q) – sedentary(Acc)	<100 counts/min	Spearman r (95 % CI) = 0.36 (0.18;0.52)	–
					Low confidence: sitting hr/day(Q) – sedentary(Acc)	<100 counts/min	Spearman r (95 % CI) = 0.45 (0.25;0.62)	–
Adults	Duncan (2001)[104]	HR (Polar)	1 weekday	7DPAR	Very hard activity(Q) – very hard activity(HR)	≥ 85 % HRR	–	MD = 0.00 hours
					Hard activity(Q) – hard activity(HR)	60-84 % HRR	–	MD = 0.02 hours
					Moderate activity(Q) – moderate activity(HR)	45-59 % HRR	–	MD = 0.21 hours
Adults	Ekelund (2006)[95]	Acc (ActiGraph)	7 days	IPAQ-s	Total MET-min/day(Q) – mean counts/min(Acc)	–	Pearson r = 0.34 (P < 0.001)	MD (95 % CI) = −25.9 (−172;120) min/day, P < 0.001
					Sitting(Q) – sedentary min/day(Acc)	<100 counts/min	Pearson r = 0.16 (P < 0.05)	–
Adults	Gauthier (2009)[105]	Ped (Yamax)	7 days	IPAQ-SALVCF	Walking(Q) – step counts(Ped)	–	Pearson r = 0.493 (P < 0.005)	–
Adults	Hagströmer (2006)[106]	Acc (ActiGraph)	7 days	IPAQ	Total MET-hr/week(Q) – total counts/min(Acc)	–	Spearman r = 0.55 (P < 0.001)	MD (95 % LoA) = 1.0 ± 16.7 hr/week
					Sitting hr/week(Q) – inactivity hr/week(Acc)	<101 counts/min	Spearman r = 0.17	–
Adults	Hagströmer (2010)[107]	Acc (ActiGraph)	7 days	IPAQ	Total min/day(Q) – total min/day(Acc)	–	Spearman r = 0.28 (P < 0.01)	–
					Total MET-min/day(Q) – total counts/min(Acc)	–	Spearman r = 0.30 (P < 0.01)	–
					Sitting min/day(Q) – sitting min/day(Acc)	<100 counts/min	Spearman r = 0.23 (P < 0.01)	MD (±SD) = 130 ± 207 min/day, P < 0.001, R^2^ = 0.50
Adults	Hallal (2010)[108]	Acc (ActiGraph)	4 days	IPAQ	Total score(Q) – total score(Acc)	≥1952 counts/min	Spearman r = 0.22	–
Adults	InterAct Consortium (2011)[51]	Acc + HR (Actiheart)	≥ 4 days	EPAQ-s	Total PA index(Q) – PAEE(Acc + HR)	–	Pearson r (95 % CI) = 0.14 (0.04;0.24), P = 0.000	–
					Cambridge index(Q) – PAEE(Acc + HR)	–	Pearson r (95 % CI) = 0.33 (0.28;0.38), P = 0.118	–
					Recreational index(Q) – PAEE(Acc + HR)	–	Pearson r (95 % CI) = 0.22 (0.16;0.28), P = 0.042	–
Adults	Jacobi (2009)[109]	Acc (ActiGraph)	7 days	MAQ	Total MET-hr/week(Q) – total counts/day(Acc)	–	Spearman r = 0.18 (P < 0.05)	–
					Sedentary hr/week(Q) – sedentary hr/week(Acc)	<100 counts/min	Spearman r = 0.14 (P < 0.1)	–
Adults	Kurtze (2008)[54]	Acc (ActiReg)	7 days	IPAQ-s	Total MET-min/week(Q) – EE(Acc)	–	Spearman r = 0.26 (P < 0.05)	MD (95 % LoA) = −433 ± 2038 min/week
					Total MET-min/week(Q) – PAL(Acc)	–	Spearman r = 0.29 (P < 0.05)	–
					Sitting hr/day(Q) – EE(Acc)	–	Spearman r = −0.25 (P < 0.05)	–
					Sitting hr/day(Q) – PAL(Acc)	–	Spearman r = −0.35 (P < 0.01)	–
Adults	Lee (2011)[98]	Acc (ActiGraph)	4 days	IPAQ-s	Total MET-min/week(Q) – total MET-min/week(Acc)	–	Spearman r (±SE) = 0.11 ± 0.03, P < 0.001	MD (±SE) = 2966.3 ± 140.1 MET-min/week, P < 0.001
					Total MET-min/week(Q) – total counts/min(Acc)	–	Spearman r (±SE) = 0.16 ± 0.03, P < 0.001	–
Adults	MacFarlane (2007)[99]	Acc (ActiGraph)	7 days	IPAQ-s	Total min/week(Q) – total MVPA min/week(Acc)	≥1952 counts/min	Spearman r = 0.09 (P = 0.52)	R^2^ = 0.78, slope = 1.59 (P < 0.01); %bias = −102, %LoA = 176
Adults	MacFarlane (2010)[110]	Acc (ActiTrainer)	7 days	IPAQ-LC	Total MET-min/day(Q) – total MET-min/day(Acc)	–	Spearman r = 0.35 (P = 0.001)	MD (95 % LoA) = −21.6 ± 575.5 MET-min/day, P = 0.643
Adults	Mahabir (2006)[89]	DLW	–	HAQ	EE(Q) – EE(DLW)	–	Spearman r = 0.36 (P < 0.05)	MD (95 % LoA) = 1782.5 ± 2237.4 kcal/day
				FCPQ	EE(Q) – EE(DLW)	–	Spearman r = 0.47 (P < 0.05)	MD (95 % LoA) = 732.8 ± 2126.7 kcal/day
				CAPS-4WR	EE(Q) – EE(DLW)	–	Spearman r = 0.16	MD (95 % LoA) = 1765.8 ± 8973.7 kcal/day
				CAPS-TWR	EE(Q) – EE(DLW)	–	Spearman r = 0.15	MD (95 % LoA) = −413.4 ± 2958.6 kcal/day
Adults	Matton (2007)[111]	Acc (RT3)	7 days	FPACQ	Employed/unemployed men: total EE(Q) – total EE(Acc)	–	Pearson r = 0.80 (P < 0.001)	*t*-test = 9.02 (P < 0.001)
					Employed/unemployed women: total EE(Q) – total EE(Acc)	–	Pearson r = 0.65 (P < 0.001)	*t*-test = 10.18 (P < 0.001)
					Retired men: total EE(Q) – total EE(Acc)	–	Pearson r = 0.55 (P < 0.01)	*t*-test = 11.48 (P < 0.001)
					Retired women: total EE(Q) – total EE(Acc)	–	Pearson r = 0.85 (P < 0.001)	*t*-test = 10.79 (P < 0.001)
					Employed/unemployed men: PAL(Q) – PAL(Acc)	–	Pearson r = 0.56 (P < 0.01)	*t*-test = 9.87 (P < 0.001)
					Employed/unemployed women: PAL(Q) – PAL(Acc)	–	Pearson r = 0.44 (P < 0.05)	*t*-test = 11.68 (P < 0.001)
					Retired men: PAL(Q) – PAL(Acc)	–	Pearson r = 0.39 (P < 0.05)	*t*-test = 11.91 (P < 0.001)
					Retired women: PAL(Q) – PAL(Acc)	–	Pearson r = 0.50 (P < 0.05)	*t*-test = 13.93 (P < 0.001)
					Employed/unemployed men: TV hr/week(Q) – TV hr/week(Acc)	–	Pearson r = 0.69 (P < 0.001)	*t*-test = −0.75
					Employed/unemployed women: TV hr/week(Q) – TV hr/week(Acc)	–	Pearson r = 0.83 (P < 0.001)	*t*-test = −3.32 (P < 0.01)
					Retired men: TV hr/week(Q) – TV hr/week(Acc)	–	Pearson r = 0.78 (P < 0.001)	*t*-test = −3.98 (P < 0.001)
					Retired women: TV hr/week(Q) – TV hr/week(Acc)	–	Pearson r = 0.80 (P < 0.001)	*t*-test = −2.41 (P < 0.05)
Adults	Nang (2011)[55]	Acc (Actical)	5 days	IPAQ	VPA(Q) – VPA(Acc)	–	Spearman r = 0.18 (P < 0.05)	MD (95 % CI) = 139 (82;196) kcal/day
					MPA(Q) – MPA(Acc)	–	Spearman r = 0.13	MD (95 % CI) = −169 (−236;-90) kcal/day
				SP2PAQ	VPA(Q) – VPA(Acc)	–	Spearman r = 0.42 (P < 0.0001)	MD (95 % CI) = 81 (47;116) kcal/day
					MPA(Q) – MPA(Acc)	–	Spearman r = 0.24 (P < 0.05)	MD (95 % CI) = −196 (−295;-97) kcal/day
Adults	Nicaise (2011)[90]	Acc (ActiGraph)	7 days	IPAQ	VPA(Q) – VPA(Acc)	≥5725 counts/min	Pearson r = −0.01	–
					MPA(Q) – MPA(Acc)	1952-5724 counts/min	Pearson r = 0.08	–
					Walking(Q) – steps(Acc)	–	Pearson r = 0.07	–
					Weekday: sitting(Q) – light PA(Acc)	≤1951 counts/min	Pearson r = −0.17	–
					Weekend: sitting(Q) – light PA(Acc)	≤1951 counts/min	Pearson r = −0.08	–
Adults	Pettee-Gabriel (2009)[91]	Acc (ActiGraph)	≥ 4 days	PMMAQ	Total MET-hr/week(Q) – total counts/day(Acc)	–	Spearman r = 0.60 (P < 0.0001)	–
					Total MET-hr/week(Q) – mean counts/min/day(Acc)	–	Spearman r = 0.59 (P < 0.0001)	–
				PWMAQ	Total MET-hr/week(Q) – total counts/day(Acc)	–	Spearman r = 0.60 (P < 0.0001)	–
					Total MET-hr/week(Q) – mean counts/min/day(Acc)	–	Spearman r = 0.56 (P < 0.0001)	–
				NHS-PAQ	Total MET-hr/week(Q) – total counts/day(Acc)	–	Spearman r = 0.46 (P < 0.001)	–
					Total MET-hr/week(Q) – mean counts/min/day(Acc)	–	Spearman r = 0.42 (P < 0.001)	–
				AAS	Total min/day(Q) – total counts/day(Acc)	–	Spearman r = 0.46 (P < 0.001)	–
					Total min/day(Q) – mean counts/min/day(Acc)	–	Spearman r = 0.50 (P < 0.0001)	–
				WHI-PAQ	Total MET-hr/week(Q) – total counts/day(Acc)	–	Spearman r = 0.47 (P < 0.001)	–
					Total MET-hr/week(Q) – mean counts/min/day(Acc)	–	Spearman r = 0.45 (P < 0.001)	–
Adults	Philippaerts (1999)[96]	DLW	14 days	BAQ	Total activity index(Q) – ADMR(DLW)	–	Pearson r = 0.68 (P < 0.01)	–
					Total activity index(Q) – PAL(DLW)	–	Pearson r = 0.69 (P < 0.001)	–
				FCPQ	7 day index(Q) – ADMR(DLW)	–	Pearson r = 0.61 (P < 0.01)	–
					7 day index(Q) – PAL(DLW)	–	Pearson r = 0.34	–
				TCQ	TEE(Q) – ADMR(DLW)	–	Pearson r = 0.63 (P < 0.01)	–
					TEE(Q) – PAL(DLW)	–	Pearson r = 0.64 (P < 0.01)	–
Adults	Philippaerts (2001)[97]	Acc (Tracmor)	4 days	BAQ	Total activity index(Q) – mean counts(Acc)	–	Pearson r = 0.47 (P < 0.001)	–
				TCQ	TEE(Q) – mean counts(Acc)	–	Pearson r = 0.22	–
Adults	Richardson (2001)[100]	Acc (Caltrac)	14x 2 days	S7DR	Men, visit 10: total MET-min/day(Q) – total MET-min/day(Acc)	–	Spearman r = 0.54 (P < 0.01)	–
					Men, visit 11: total MET-min/day(Q) – total MET-min/day(Acc)	–	Spearman r = 0.45 (P < 0.05)	–
					Women, visit 10: total MET-min/day(Q) – total MET-min/day(Acc)	–	Spearman r = 0.20	–
					Women, visit 11: total MET-min/day(Q) – total MET-min/day(Acc)	–	Spearman r = 0.06	–
Adults	Saglam (2010)[112]	Acc (Caltrac)	4 days	IPAQ	Total MET-min/week(Q) – TEE(Acc)	–	Spearman r (95 % CI) = 0.29 (0.05;0.47), P = 0.009	–
				IPAQ-s	Total MET-min/week(Q) – TEE(Acc)	–	Spearman r (95 % CI) = 0.30 (0.07;0.49), P = 0.008	–
Adults	Schmidt (2006)[92]	Acc (ActiGraph)	7 days	KPAS-mod	Total activity score(Q) – mean counts/min(Acc)	–	Spearman r = 0.52	–
					Weighted activity score(Q) – mean counts/min(Acc)	–	Spearman r = 0.59	–
Adults	Smitherman (2009)[113]	Acc (ActiGraph)	1 day	JPAC	JPAC total score(Q) – mean counts/min(Acc)	–	Spearman r = 0.24 (P < 0.0001)	–
Adults	Staten (2001)[93]	DLW	8 days	AAFQ	TEE-ic(Q) – TEE(DLW)	–	Pearson r = 0.40 (P < 0.001)	MD = 1935 kJ/day
					TEE-mif(Q) – TEE(DLW)	–	Pearson r = 0.45 (P < 0.001)	MD = 697 kJ/day
					TEE-met(Q) – TEE(DLW)	–	Pearson r = 0.58 (P < 0.001)	MD = 3595 kJ/day
Adults	Strath (2004)[114]	Acc + HR (ActiGraph + Polar)	7 days	CAQ-PAI	MET-min/week(Q) – MET-min/week(Acc + HR)	–	Spearman r = 0.35	–
Adults	Trinh (2009)[115]	Acc (ActiGraph)	7 days	GPAQ	Dry season: GPAQ total score(Q) – total counts(Acc)	–	Spearman r = 0.33	MD (95 % LoA) = 2.6 (0.03;224)
					Wet season: GPAQ total score(Q) – total counts(Acc)	–	Spearman r = 0.19	MD (95 % LoA) = 2.6 (0.03;224)
					Dry season: sedentary time(Q) – sedentary time(Acc)	<100 counts/min	Spearman r = 0.22	–
					Wet season: sedentary time(Q) – sedentary time(Acc)	<100 counts/min	Spearman r = 0.31	–
Adults	Washburn (2003)[116]	DLW	14 days	S7DR	TEE(Q) – TEE(DLW)	–	Pearson r = 0.58 (P < 0.01)	MD (95 % LoA) = −96 ± 4161 kJ/day
					PAEE(Q) – PAEE(DLW)	–	Pearson r = 0.12	MD (95 % LoA) = −222 ± 4144 kJ/day
Adults	Wolin (2008)[117]	Acc (Actical)	6 days	IPAQ-s	1-min bout: MET-min/week(Q) – counts/day(Acc)	–	Spearman r = 0.36 (P < 0.001)	κ (95 % CI) = 0.21 (−0.04;0.47)
					10-min bout: MET-min/week(Q) – counts/day(Acc)	–	Spearman r = 0.26 (P = 0.002)	κ (95 % CI) = 0.04 (0.01;0.06)
Elderly	Bonnefoy (2001)[75]	DLW	14 days	MLTPAQ	Total activity(Q) – TEE(DLW)	–	Pearson r = 0.23, Spearman r = 0.17	–
				YPAS	Summary index(Q) – TEE(DLW)	–	Pearson r = 0.11, Spearman r = 0.10	–
				BAQ-mod	Questionnaire score(Q) – TEE(DLW)	–	Pearson r = 0.21, Spearman r = 0.28	–
				CAQ	Total activity(Q) – TEE(DLW)	–	Pearson r = 0.39, Spearman r = 0.37	–
				7DR	Total activity(Q) – TEE(DLW)	–	Pearson r = 0.37, Spearman r = 0.51 (P < 0.05)	–
				DQ-mod	Total score(Q) – TEE(DLW)	–	Pearson r = 0.21, Spearman r = 0.34	–
				LRC	Enhanced LRC score(Q) – TEE(DLW)	–	Pearson r = 0.33, Spearman r = 0.29	–
				SUA	MPA(Q) – TEE(DLW)	–	Pearson r = 0.65 (P < 0.05), Spearman r = 0.46	–
					VPA(Q) – TEE(DLW)	–	Pearson r = 0.63 (P < 0.05), Spearman r = 0.64 (P < 0.05)	–
				PASE	Total score(Q) – TEE(DLW)	–	Pearson r = 0.28, Spearman r = 0.23	–
				QAPSE	Mean habitual DEE(Q) – TEE(DLW)	–	Pearson r = 0.32, Spearman r = 0.25	–
Elderly	De Abajo (2001)[76]	Acc (Caltrac)	3 days	YPAS	Total hr/week(Q) – activity units/day(Acc)	–	Pearson r = 0.20 (P = 0.049)	–
					TEE(Q) – activity units/day(Acc)	–	Pearson r = 0.23 (P = 0.022)	–
					YPAS summary index(Q) – activity units/day(Acc)	–	Pearson r = 0.24 (P = 0.018)	–
					Sitting(Q) – activity units/day(Acc)	–	Pearson r = −0.06 (P = 0.54)	–
Elderly	Dinger (2004)[77]	Acc (ActiGraph)	7 days	PASE	Total PASE score(Q) – mean counts/min(Acc)	–	Spearman r = 0.43 (P = 0.001)	–
Elderly	Dubbert (2004)[78]	Acc (Tritrac R3D)	3 days	7DPAR	TEE(Q) – counts/min(Acc)	–	Spearman r = 0.49 (P < 0.01)	–
Elderly	Giles (2009)[79]	Ped (Yamax)	7 days	CHAMPS-MMSCV	Volume T1: walking(Q) – step counts(Ped)	–	Spearman r = 0.40 (P < 0.01)	–
					Frequency T1: walking(Q) – step counts(Ped)	–	Spearman r = 0.57 (P < 0.01)	–
					Volume T2: walking(Q) – step counts(Ped)	–	Spearman r = 0.53 (P < 0.01)	–
					Frequency T2: walking(Q) – step counts(Ped)	–	Spearman r = 0.60 (P < 0.01)	–
Elderly	Hagiwara (2008)[80]	Acc (Kenz Lifecorder)	3 days	PASE	Total PASE score(Q) – EE(Acc)	–	Spearman r = 0.16 (P = 0.02)	–
					Total PASE score(Q) – walking steps(Acc)	–	Spearman r = 0.17 (P = 0.01)	–
Elderly	Harada (2001)[81]	ML (Mini-Mitter)	7 days	CHAMPS	EE(Q) – ankle counts(ML)	–	Pearson r = 0.36 (P < 0.01)	–
					EE(Q) – waist counts(ML)	–	Pearson r = 0.42 (P < 0.001)	–
				PASE	Total PASE score(Q) – ankle counts(ML)	–	Pearson r = 0.59 (P < 0.001)	–
					Total PASE score(Q) – waist counts(ML)	–	Pearson r = 0.52 (P < 0.001)	–
				YPAS	EE(Q) – ankle counts(ML)	–	Pearson r = 0.46 (P < 0.001)	–
					EE(Q) – waist counts(ML)	–	Pearson r = 0.61 (P < 0.001)	–
Elderly	Hurtig-Wennlöf (2010)[82]	Acc (ActiGraph)	7 days	IPAQ-E	Walking + MPA min/day(Q) – mean counts/min(Acc)	–	Spearman r = 0.347 (P < 0.01)	κ (95 % CI) = 0.448 (0.18;0.72), P < 0.001
					VPA min/day(Q) – VPA counts/min(Acc)	>4944 counts/min	Spearman r = 0.369 (P < 0.01)	–
					MPA min/day(Q) – MPA counts/min(Acc)	760-4944 counts/min	Spearman r = 0.396 (P < 0.01)	–
					Sitting min/day(Q) – sitting counts/min(Acc)	<100 counts/min	Spearman r = 0.277 (P < 0.05)	–
Elderly	Kolbe-Alexander (2006)[83]	Acc (ActiGraph)	7 days	IPAQ-s	Men: vigorous MET-min/week(Q) – high counts(Acc)	≥5725 counts/min	Spearman r = 0.43 (P = 0.05)	–
					Women: vigorous MET-min/week(Q) – high counts(Acc)	≥5725 counts/min	Spearman r = 0.05	–
					Men: moderate MET-min/week(Q) – moderate min(Acc)	1952-5724 counts/min	Spearman r = 0.31 (P = 0.004)	–
					Women: moderate MET-min/week(Q) – moderate min(Acc)	1952-5724 counts/min	Spearman r = −0.09	–
					Men: walking MET-min/week(Q) – total counts(Acc)	–	Spearman r = 0.57 (P = 0.00007)	–
					Women: walking MET-min/week(Q) – total counts(Acc)	–	Spearman r = 0.42 (P = 0.006)	–
					Men: sitting MET-min/week(Q) – total counts(Acc)	–	Spearman r = −0.40 (P = 0.001)	–
					Women: sitting MET-min/week(Q) – total counts(Acc)	–	Spearman r = −0.35 (P = 0.005)	–
				YPAS	Men: total MET-min/week(Q) – total counts(Acc)	–	Spearman r = 0.54 (P = 0.0002)	–
					Women: total MET-min/week(Q) – total counts(Acc)	–	Spearman r = 0.13	–
Elderly	Starling (1999)[84]	DLW	10 day	MLTPAQ	Men: TEE(Q) – TEE(DLW)	–	–	MD (95 % LoA) = 752 ± 972 kcal/day
					Women: TEE(Q) – TEE(DLW)	–	–	MD (95 % LoA) = 487 ± 698 kcal/day
				YPAS	Men: TEE(Q) – TEE(DLW)	–	–	MD (95 % LoA) = 104 ± 1414 kcal/day
					Women: TEE(Q) – TEE(DLW)	–	–	MD (95 % LoA) = 9 ± 972 kcal/day
Elderly	Tomioka (2011)[85]	Acc (Kenz Lifecorder)	2 weeks	IPAQ-s	Young old men: MET-min/week(Q) – MET-min/week(Acc)	–	Spearman r = 0.42 (P < 0.01)	κ (95 % CI) = 0.49 (0.34;0.64)
					Young old women: MET-min/week(Q) – MET-min/week(Acc)	–	Spearman r = 0.49 (P < 0.01)	κ (95 % CI) = 0.39 (0.22;0.56)
					Old old men: MET-min/week(Q) – MET-min/week(Acc)	–	Spearman r = 0.53 (P < 0.01)	κ (95 % CI) = 0.46 (0.29;0.63)
					Old old women: MET-min/week(Q) – MET-min/week(Acc)	–	Spearman r = 0.49 (P < 0.01)	κ (95 % CI) = 0.47 (0.28;0.66)
Elderly	Washburn (1999)[86]	Acc (ActiGraph)	3 days	PASE	Total PASE score(Q) – mean counts/5 min epoch(Acc)	–	Spearman r = 0.49 (P < 0.05)	–
							*Median Spearman r = 0.30 (youth: 0.25, adults: 0.30, elderly: 0.40)*	
							*Median Pearson r = 0.39 (youth: 0.38, adults: 0.46, elderly: 0.345)*	

Q1 = first completed questionnaire, Q2 = second completed questionnaire, Q3 = third completed questionnaire, r = correlation coefficient (rho), CI = Confidence Interval (lower;upper), κ = kappa (i.e. Cohen weighted kappa unless specified otherwise), LoA = Limits of Agreement, MD = Mean Difference, – = not stated.

Acc = Accelerometry [NB: ActiGraph (Model 7164) is successor of preceding accelerometer by MTI, formerly CSA]. Accelerometer names as used in the respective papers.

Affuso (2011): Sedentary mins = total minutes TV/video watching, computer/internet use, talking on phone, playing video/computer games.

Ainsworth (1999): MPA MET-min/week = energy expended in moderate-intensity occupational standing activities. 7DR-scores = scores of occupational activity only. EE = Energy Expenditure in kcal/day. All other associations between the TOQ and Caltrac scores were low and non significant.

Allor (2001): HR monitor brand not specified. EE = Energy Expenditure in kcal/hr.

Bonnefoy (2001): MLTPAQ total activity = light, moderate, heavy, household activity. YPAS summary index = sum of vigorous, walking, moving, standing, sitting scores. BAQ-mod questionnaire score = sum of household, sports, leisure activity scores. CAQ total activity = sum of walking, stairs, sports. 7DR total activity = weighted sum of sleep, light, moderate, hard, very hard activity. Dallosso-mod total score = weighted sum of walking standing, productive, leisure, muscle-loading activity. Enhanced LRC score = self report of usual activity. SUA MPA = six habitual moderate activities. SUA VPA = five habitual vigorous activites. PASE total score = activity weight*frequency across work-related leisure, household activities. QAPSE mean habitual DEE = activity weight*duration as daily energy expenditure.

Brown (2008): Frequency/week = frequency of total activity per week. Total min/week = minutes per week of total activity ≥3 METs. Total counts = all accelerometer recorded minutes.

Bull (2009): VPA/MPA = total vigorous/moderate intensity activity across all domains. Sedentary min/day = time spent sitting per day in minutes. Data categorized for studies in China (n = 215) and South Africa (n = 83).

Conway (2002): R^2^ = regression against PAR; explained variance is 10 % for 7DPAR and 14 % for S7DR. MD = mean differences ± SEM (percentages in parentheses) between each method and EE(DLW).

Cust (2008): Total MET-hr/week = total MET hours per week of non-occupational activity. Total PA index = cross-tabulation of level of occupational activity with combined recreational and household activities - inactive, moderately inactive, moderately active, active. Cambridge PA index = index based on occupational, cycling and sports activity (generally more intense activities).

Cust (2009): Results are stratified according to the group of participants reporting high or low confidence in recall of PA. High confidence = group of participants reporting high self-reported confidence in recall of physical activity. Low confidence = group of participants reporting low self-reported confidence in recall of physical activity. Remarkably, the correlation for the Cambridge index is slightly higher compared to the total PA index (MET-hrs) comparing accelerometry with the EPAQ. Total MET-hr/week(Acc) = total physical activity in MET-hr/week, calculated as light + moderate + vigorous activity (no sedentary time). Data are averages of three 7-day accelerometer periods.

De Abajo (2001): Total hr/week = total activity time. Activity units = kilocalorie score divided by resting metabolic rate. TEE = Total Energy Expenditure in kJ/day. YPAS summary index = summed time for each activity, expressed in hours per week for each subject. Individual indices were created by multiplying a frequency score by a duration score and multiplying again by a weighting factor.

Dinger (2004): Total PASE score = weighted and summed score of individual items using the PASE scoring algorithm.

Duncan (2001): HRR = each subject's individual heart rate reserve (individual maximal MET capacity), where HRmax was determined from the graded exercise test and HRrest from the average of three measures after a 10-min seated test. Mean difference = 0.21, i.e. 0.21 hours overreported in PAR.

Eisenmann (2002): Total leisure activity score was calculated by multiplying the frequency of each category by the MET value and summing the score.

Ekelund (2005): MD = mean difference between objectively measured accelerometry time in MVPA and self-reported time in MVPA and walking.

Giles (2009): Volume T1/T2 = walking MET-min per week at first/second administration (T1/T2) of the CHAMPS. Frequency T1/T2 = walking sessions per week at first/second administration (T1/T2) of the CHAMPS.

Hagiwara (2008): PASE score was calculated by adding the score for each component determined on the basis of the time spent on each activity or the presence or absence of activity over the past 7 days. EE = Energy Expenditure divided by bodyweight in kcal/day/wt. Walking steps = daily number of walking steps measured by the Lifecorder accelerometer.

Hagströmer (2008): Data shown is data from the average intensity measured by the accelerometer.

Hagströmer (2006): Bland-Altman results from analysis for time spent in at least moderate physical activity (hr/week) as assessed by the IPAQ and measured using an activity monitor.

Hallal (2010): Total score(Q) = sum of minutes spent on MPA (including walking) per week, and twice the number of minutes spent on VPA, calculated from the IPAQ data. Total score(Acc) = accelerometer-based total score: moderate + vigorous-intensity counts.

Harada (2001): MiniLogger measures activity by counting the number of mercure switch closures, resulting in a 'count' of activity, over a predetermined time interval. EE = Energy Expenditure in kcal/week. Total PASE score = total score computed by 1) multiplying an activity frequency value from a conversion of hours per day in six categories of activity (e.g., moderate sports) by the respective weight and summing over these activities and 2) adding a weight to this summated score for each six other household activities if the activity was reported over the past 7 days.

Huang (2009): Results from Bland-Altman analysis are combined results for boys and girls (no results for sedentary time). Cut points used are Freedson age-based cut point, calculated as METs = 2.757 + (0.0015*counts per minute) - (0.0896*age[yr]) - (0.000038*counts per minute*age[yr]).

Hurtig-Wennlöf (2010): Agreement (κ) = Cohen's kappa for testing total agreement between the IPAQ-E and accelerometry.

InterAct Consortium (2011): Total PA index = cross-tabulation of level of occupational activity with combined recreational and household activities (MET-hr/week) - inactive, moderately inactive, moderately active, active. Cambridge index = index based on occupational, cycling and sports activity (h/week). Recreational index = index based on quartiles of the sum of walking, cycling, and sports (MET-hr/week). Fisher-transformed correlations were estimated for each country, and random effect meta-analysis methods were used to calculate the overall combined correlation of PAEE (kJ/kg/day) measured by the combined HR and movement sensor with the three PA indices from the EPAQ-s.

Jacobi (2009): Sedentary time = time spent watching TV/video or playing video games and time spent using a computer.

Kolbe-Alexander (2006): High counts = counts in high-intensity physical activity. Moderate min = time spent in moderate-intensity activity. Total counts = total counts for physical activity. Sitting = time spent sitting during a weekend day.

Kowalski (1997): PAQ-C score = calculated as the mean of the nine items, ranging from 1 to 5. Total counts = total counts measured by the Caltrac that reflect vertical acceleration of the body.

Kurtze (2008): EE = Energy Expenditure in MJ/day. PAL = average Physical Activity Level in 7 days, calculated as total EE divided by basal metabolic rate (BMR). Results from Bland-Altman analysis are combined results for total moderate, vigorous and walking activity.

MacFarlane (2007): Total MVPA min/week(Q) = total weighted minutes, calculated as moderate + (2*vigorous). R^2^, slope = result from regression analysis between the Bland-Altman differences and averages. %Bias, LoA = bias and limits of agreement expressed as percentage of the mean score.

Mahabir (2006): Duration of validation not stated, likely to be 14 days. EE = Energy Expenditure in kcal/day.

Martinez-Gomez (2010): Correlation coefficient = correlation between the two instruments for the 3 day mean.

Martinez-Gomez (2011): PAQ-A score = mean score of 8 activity items scored on a 5-point scale.

Matton (2007): EE = Energy Expenditure in kcal/week. PAL = Physical Activity Level, calculated as total EE divided by 168 (number of hours per week) and the reported body weight. TV hr/week = time per week spent watching television or videos or playing computer games; this time was recalled in the FPACQ and also directly coded in the written activity log of the accelerometer reflecting the same activity domain. T-test = paired t-test to compare the magnitude of activity variables calculated from the RT3 and FPACQ (absolute validity).

Nang (2011): VPA(Q) = 3–6 METs kcal/day, MPA(Q) = >6 METs kcal/day. VPA(Acc), MPA(Acc) = moderate and vigorous physical activity using cutoff points of 3 METs between light and moderate activity, and 6 METs between moderate and vigorous activity.

Nicaise (2011): PA variables from questionnaire assessed in MET-min/week. Steps(Acc) = number of steps taken per day (from the dual mode function).

Pettee-Gabriel (2009): Participants wore the accelerometer on average 6.3 ± 0.7 days/week or 30.7 ± 4.8 days during 35 days of observation and 14.4 ± 1.1 hours/day.

Philippaerts (1999): Total activity index = index calculated from the work, sport and leisure time index. ADMR = Average Daily Metabolic Rate in MJ/day. PAL = Physical Activity Level, determined as the ratio of ADMR (Average Daily Metabolic Rate) over SMR (Sleeping Metabolic Rate). 7 day index = index in kcal/day calculated from hours spent on vigorous (8 times resting metabolic rate) and moderate (4 times resting metabolic rate) activities and including sleeping time and the time spent on light activities (remaining time) during the last seven days. TEE = Total Energy Expenditure in kcal/day.

Philippaerts (2001): Total activity index = index calculated from the work, sport and leisure time index. TEE = Total Energy Expenditure in kcal/day.

Rangul (2008): Frequency = out of breath or sweat sessions per week. Duration = out of breath or sweat hours per week. TEE = Total Energy Expenditure in MJ/week. PAL = Average Physical Activity Level for 7 days, calculated as total energy expenditure divided by basal metabolic rate.

Richardson (2001): Visit 10/11 = comparison for direct validation at study visit 10/11. Caltrac MET-min/day are obtained by dividing average 24-hour Caltrac readings (kcal/day) by the Caltrac's estimate of 24-hour resting energy expenditure and multiplying by 1440 min/day.

Scerpella (2002): 2x 3 Days = two measurement periods of 2 weekdays and 1 weekend day. Score calculations not specifically reported.

Schmidt (2006): Total activity score = activity score of all four domains, calculated as: (household/caregiving index*0.25 + occupational index*0.25 + active living index*0.25 + sports/exercise index*0.25)*4. Counts/min = mean accelerometer output per 1-min epoch, reflecting raw accelerometer output without any categorization according to activity intensity. Weighted activity score = activity score of all four domains, calculated as: (household/caregiving index*0.50 + occupational index*0.20 + active living index*0.25 + sports/exercise index*0.05)*4.

Slinde (2003): eMLTPAQ = extended MLTPAQ with additional questions about inactivity during leisure time. TEE = Total Energy Expenditure in MJ/day. Sedentary min/day = time spent watching TV, videos and computer time.

Smitherman (2009): JPAC total score = total score calculated by summing the 4 index scores (active living, work, home/family/yard/garden, sport/exercise index) and can range from 3 to 20.

Starling (1999): TEE = Total Energy Expenditure in kcal/day.

Staten (2001): TEE = Total Energy Expenditure in kJ/day, -ic = average total energy expenditure with RMR measured by indirect calorimetry, -mif = average total energy expenditure with RMR calculated using the Mifflin et al. Equation, -met = average total energy expenditure with RMR calculated using the MET conversion.

Tomioka (2011): Young old = age 65–74, old old = age 75–89.

Treuth (2004): GAQ score yesterday = summary score estimated from 18 physical activities reliably recalled and frequently performed on the previous day (yesterday) or usually. The GAQ summary scores were computed as the total MET-weighted score divided by the number of nonmissing items. Average counts/min: all counts measured between 6 AM to 12 midnight averaged per minute. Baseline: n = 197, follow-up: n = 168.

Trinh (2009): Dry season is baseline (n = 135). Measurements in wet season (n = 116) were performed 2 months after baseline during dry season. Sedentary time = time spent sitting or reclining. Mean (95 % LoA) = log-transformed average difference between the time spent in MVPA measured with GPAQ (averaged over dry and wet season) and accelerometer with 95 % limits of agreement.

Troped (2007): MPA = number of days participating in ≥ 30 min of moderate PA during past 7 days. VPA = number of days participating in ≥ 20 min of vigorous PA during past 7 days. Sensitivity = probability of the YRBS items correctly classifying students as meeting recommendations. Specificity = probability of YRBS items correctly classifying students as not meeting the recommended level of PA. Kappa range = range of kappa coefficients between Actigraph measures (accumulated minutes, minutes in bouts ≥ 5 min, minutes in bouts ≥ 10 min, sustained minutes of PA) and the YRBS measure. Cut points used are based on the Freedson age-dependent equation; METs = 2.757 + (0.0015*counts per minute) - (0.0896*age[yr]) - (0.000038*counts per minute*age[yr]).

Washburn (1999): Total PASE score was computed by multiplying the amount of time spent in each activity (hours/week) or participation (yes/no) in an activity by the empirically derived item weights and summing over all activities. Accelerometer readings are averaged over five-minute epoch periods.

Washburn (2003): Interviewer reliability tested: ICC = 0.85. TEE = Total Energy Expenditure, including sleep, in kJ/day. PAEE = Physical Activity Energy Expenditure, i.e. light, moderate, hard and very hard activities, excluding sleep.

Weston (1997): 1 Day = 1 day after school hours. TEE = Total relative Energy Expenditure in kcal/kg/day. EE = mean estimated rate of Energy Expenditure in kcal/kg/hr for the entire after school period, derived from both mode and intensity. %HRR = mean percent of heart rate range. HRR was calculated as HRmax - HRrest, where HRmax was estimated from the formula 220 - age, and HRrest was taken from the mean of the five lowest 1-min heart rates recorded during the measurement period. All heart rates (HRraw) were converted to a %HRR using the formula HRraw/HRR*100 and averaged to produce mean %HRR.

Wolin (2008): 1-Min bout = accelerometer bout lasting at least 1 minute. 10-Min bout = accelerometer bout lasting at least 10 minutes.

Of the 65 studies that report new results for the validity of existing questionnaires, 14 studies [55,61,69,75,81,83,84,87,89,91,94,96,97,103] tested two or more questionnaires. Forty-five studies used accelerometry as the criterion, and the remaining used DLW (n = 8) [71,75,84,89,93,94,96,116], pedometry (n = 3) [79,101,105], HR monitoring (n = 1) [104], MiniLogger (n = 1) [81] or a combination of methods (n = 5) [51,60,61,74,114]. Spearman and Pearson correlations were the most commonly used statistical measures for assessing validity; four studies reported 95 % confidence intervals with these correlations [51,102,103,112] and three studies solely reported results using the Bland-Altman levels of agreement method [84,94,104]. Median correlations between reported sedentary behaviours and inactivity from objective measures were calculated: Spearman r = 0.23, Pearson r = 0.435.

### Youth

Median validity correlations for the youth were as follows: Spearman r = 0.25, Pearson r = 0.38. Many PAQs (SAPAC [59], HBSC [54], IPAQ-s [54], GSQ [70] and GAQ [118]) demonstrated low validity coefficients (r < 0.2) in youth and only one instrument (PDPAR [60]) was regarded as highly valid (r = 0.76) when compared with physical activity assessed by the Caltrac accelerometer.

### Adults

Median validity correlations for adults were as follows: Spearman r = 0.30, Pearson r = 0.46. Validity correlations were generally low for most PAQs, except for the FPACQ [111] compared with accelerometry in multiple subcategories (r = 0.39–0.85) and the BAQ (r = 0.68–0.69), FCPQ (r = 0.34–0.61) and TCQ (r = 0.63–0.64) for estimated TEE compared with TEE measured with the DLW method [96]. Pettee-Gabriel et al. compared five different PAQs with accelerometry from the Actigraph accelerometer and showed acceptable validity for all instruments; PMMAQ (r = 0.59–0.60), PWMAQ (r = 0.56–0.60), NHS-PAQ (r = 0.42–0.46), AAS (r = 0.46–0.50), WHI-PAQ (r = 0.45–0.47) [91]. Several studies, including the 7DR-O [87], MAQ [109], CAPS [89], IPAQ [55,90] and the IPAQ-s [54,98,99], demonstrated poor validity.

### Elderly

Median validity correlations for the elderly were as follows: Spearman r = 0.40, Pearson r = 0.345. Bonnefoy et al. tested the validity of 10 previously developed well known PAQs using DLW as the criterion measure [75]. The results of this study suggested that the Stanford Usual Activity questionnaire performed best (r = 0.63–0.65). Other studies in elderly generally found low correlations between self-reported PA with objective measures, also demonstrated by the generally weak performances of the YPAS in several studies (r = 0.11–0.61) [75,76,81,83,84], and PASE in one of the studies (r = 0.16–0.17) [80].

## Discussion

This systematic review covered the most recent 15-year period. We identified 31 studies that adequately tested newly developed PAQs for both validity and reliability during this period. This suggests that whilst assessing physical activity by means of objective monitoring has become widespread also when examining population levels of activity [119-121], PAQs remain an active area of research and are now generally considered complementary to any objective measure. Several previous reviews have assessed the reliability and validity of PAQs with a special focus on their overall performance [9], or performance in specific age groups [11,14,15]. Conversely, we compared whether newly developed PAQs performed better than older PAQs, as this will inform researchers and practitioners when choosing an existing PAQ or developing a new instrument for assessing physical activity. We therefore comprehensively summarized the results to allow an adequate appraisal of the existing PAQs performance across domains and physical activity intensities.

In concordance with previous reviews [11,14,15], very few questionnaires showed acceptable reliability and validity across age groups. Developing new PAQs requires careful consideration of the study design in terms of target population, sample size, age group, recall period, dimension and intensity of PA, relative and absolute validity, standardized quality criteria and appropriate comparison measures. The lack of formulating a priori hypotheses was recently highlighted as a limitation in most studies examining the validity of PAQs [11] and comprehensive key criteria for physical activity and sedentary behaviour validation studies have been proposed [122,123].

Since the comprehensive review by Kriska and Caspersen [9], it is apparent that more appropriate criterion methods, in particular accelerometry, have been used to test the validity of PAQs. Yet, a considerable number of studies were excluded from the present review due to an inappropriate criterion method (e.g. aerobic fitness). Many studies reported reliability and validity results for existing and well established questionnaires, which suggests that these instruments are still frequently used. Importantly, newly developed PAQs do not seem to perform any better than existing instruments in terms of reliability and validity. Unfortunately, we were not able to conduct a formal meta-analysis due to differences in reported outcomes, different criterion measures and different time frames between questionnaires.

Total energy expenditure (TEE) was frequently used as the outcome measure of the PAQ and the validity scores from these types of instruments are usually high. However, the results from many of these studies should be interpreted carefully. This is because TEE from any self-report incorporates an estimate of resting energy expenditure (REE) generally calculated from body weight, sex and age. REE explains most of the variation in TEE and, consequently, high correlations may be generated when comparing TEE from self-report with measured or estimated TEE from the criterion method. This is particularly problematic when those same predictions of REE are used by both the criterion method and the self-reported calculation of energy expenditure. Therefore, other outputs (e.g. time spent in different intensity levels, physical activity energy expenditure normalised for body size) from the criterion method appear more appropriate to serve as criterion measures. In these studies correlations between the criterion measure and self-reported PA are considerably weaker than those for TEE, although the concerning PAQs may still be considered valid as demonstrated in some studies [31,116]. The notion of validity, however, is a matter of degree, rather than an all-or-nothing determination.

The validity correlation coefficients from the vast majority of existing and newly developed PAQs were considered poor to moderate and usually only acceptable when results were presented as Pearson or Spearman correlation coefficients. This suggests that most PAQs may be valid for ranking individuals’ behaviour whereas their absolute validity is limited to quantify PA. Although our summary of the correlations in a single median value should be interpreted with caution, we did not observe any substantial difference between newly and existing PAQs. This may suggest that, despite considerable effort, accurate and precise self-report physical activity instruments are still scarce [124]. Many of the newly developed instruments collected information in various domains of physical activity including transportation and housework. Despite this, it appears almost impossible to obtain a valid estimation of a highly variable behaviour such as free-living physical activity by self-report. While results from large scale observational cohort studies have convincingly demonstrated the beneficial effects of self-reported physical activity on various health outcomes including all-cause mortality, coronary and cardiovascular disease morbidity and mortality, some types of cancer, and type 2 diabetes, the detailed dose–response associations are still unknown [125]. Increased sample size is usually considered to improve precision but may not overcome issues about accuracy. Further, a large sample size does not overcome misclassification due to differential measurement error. Therefore, future studies should consider including an objective measure of physical activity in addition to self-report or consider recommendations to reduce self-report error [126].

With few exceptions, most PAQs reviewed showed acceptable to good reliability with only minor differences between existing and newly developed PAQs. The median reliability correlations were acceptable to good in youth (0.64 – 0.65), adults (0.64 – 0.79), and the elderly (0.60 – 0.65) for existing PAQs; and marginally higher for newly developed PAQs in youth (0.69 – 0.80), adults (0.74 – 0.765), and the elderly (0.70). However, only 3 of 11 newly developed PAQs [21,23,24] showed consistently good reliability.

For existing PAQs, median validity correlations were poor to acceptable in youth (0.25 – 0.38), adults (0.30 – 0.46), and elderly (0.345 – 0.40); and essentially similar for newly developed PAQs in youth (0.22 – 0.41), adults (0.27 – 0.28), and the elderly (0.41).

Only four of the reviewed questionnaires, the IPAQ-s (existing) [85], the FPACQ (existing) [111], PDPAR (existing) [60] and the RPAR (new) [21] showed acceptable to good results for both reliability and validity. Sedentary behaviour appeared to be one of the most difficult domains to assess with questionnaires as demonstrated by the poor correlations with objectively measured sedentary time, although arguably, there are also limitations of the criterion measures, which contribute to poorer agreement between methods. About one third (n = 11) of the studies reporting data on newly developed PAQs assessed both validity and reliability for sedentary behaviour. 17 and 15 studies reported data on validity and reliability for sedentary behaviour from existing PAQs, respectively.

Accuracy of PA recall may be increased at the second retest administration by an increased physical activity awareness as a result of completing the questionnaire previously [105]. Many of the reviewed studies did not specify details about their reliability testing, making it difficult to distinguish test-retest reliability of the instrument from a measure of stability of physical activity. It is therefore complex to assign the correlations to either the reliability of the instrument or to the stability of the behaviour of the participant. Assessing test-retest reliability for a last seven day PAQ is generally more straight forward compared to a PAQ assessing usual or last year physical activity. This is because when examining the reliability of a last seven days instrument the respondents should be prompted to report their PA during exactly the same week at two different occasions separated in time. However, this must be weighed against administering the test and retest too close in time that the respondent remembers the answers given to the first administration, resulting in inflation of reliability estimates from correlated error. Several other study details than timeframe of recall can be identified to have a marked influence on the study results, such as socio-cultural background, sex, age, literacy, and cognitive abilities.

The DLW method is usually considered the most accurate criterion method available for measuring TEE and PAEE. However, as discussed above, when using the DLW method and other objective methods which provide outputs in TEE as the criterion instrument, individual variability in body weight needs to be considered. It is therefore recommended that data from these methods should be expressed as PAEE, with and without normalisation for body weight in subsequent validation studies. Combined heart rate and movement sensing may be more accurate than either of the methods used alone for measuring time spent at different intensity levels [31]. However, most of the newly developed PAQs used a single accelerometer mounted at the hip as the criterion method, possibly due to its reasonable costs and feasibility in large study groups. Accelerometry also has some inherent limitations including its inability to accurately assess the intensity of specific types such as weight-bearing activities, cycling, and swimming [33]. Further, the choice of somewhat arbitrary cut-off points [127-129] to classify intensities of activity when using accelerometry as a criterion method has been documented before. The use of accelerometers is especially problematic to validate time spent in different intensities of physical activity from PAQs and this also hampers comparison of studies [33]. Usually criterion measures assess overall PA (e.g. time in MVPA, PAEE) which precludes a direct test of the validity of self-reported domain specific activity (e.g. occupation). It is therefore not surprising that some PAQs [e.g. 86] which only asses a specific domain of activity demonstrate low validity when compared with overall physical activity from the criterion instrument. More research is therefore needed to compare time stamped criterion data with domain specific self-reported activity and to develop criterion instruments which can accurately categorise types of activities. Adopting a conceptual framework for physical activity [130] in combination with standardized procedures when developing and validating PAQs [122,123] is highly recommended.

Pearson and Spearman correlations may not be the most appropriate statistical methods to use for reporting results on the validity of PAQs. ICC is considered a more appropriate method for continuous measures on the same scale, whereas weighted kappa is a better choice of method for categorical measures [131,132]. When reporting validation results researchers are encouraged to report absolute validity in terms of mean bias with limits of agreement as well as the error structure of the instrument across the measurement range. We noted that many of the newly developed instruments reported results on absolute validity by means of the Bland-Altman method, which is a simple, intuitive and easy to interpret method to analyse assess measurement error [133]. Descriptive details of the study population may be helpful to explain any heterogeneity in the findings from different studies. Researchers can individually interpret all data for quality and applicability.

In summary, we systematically reviewed studies assessing both reliability and validity of PAQs in various domains, across age groups, and with a focus on total PA and sedentary time. PAQs are inherently subject to many limitations and the choice of PAQs should be dictated by the research question and the population under study. Considerations for researchers when using PAQs in practice have been identified and new research should consider including an objective method for assessing physical activity in addition to any self-report [134]. This review has identified a limited number of PAQs that appear to have both acceptable reliability and validity. Newly developed PAQs do not appear to perform substantially better than existing PAQs in terms of reliability and validity.

## Competing interest

The authors declare they have no competing interest to declare.

## Authors’ contribution

HH performed an updated literature search and drafted the manuscript. SB contributed to the design of the study and critically revised the manuscript. JW and HB contributed to the design of the study and performed the original literature search.UE contributed to the design of the study, contributed to the literature search and solved issues about inclusion of manuscripts, and critically revised the manuscript.All authors approved the final version of the manuscript.
